# High economic inequality is linked to greater moralization

**DOI:** 10.1093/pnasnexus/pgae221

**Published:** 2024-06-05

**Authors:** Kelly Kirkland, Paul A M Van Lange, Drew Gorenz, Khandis Blake, Catherine E Amiot, Liisi Ausmees, Peter Baguma, Oumar Barry, Maja Becker, Michal Bilewicz, Watcharaporn Boonyasiriwat, Robert W Booth, Thomas Castelain, Giulio Costantini, Girts Dimdins, Agustín Espinosa, Gillian Finchilescu, Ronald Fischer, Malte Friese, Ángel Gómez, Roberto González, Nobuhiko Goto, Peter Halama, Camilo Hurtado-Parrado, Ruby D Ilustrisimo, Gabriela M Jiga-Boy, Peter Kuppens, Steve Loughnan, Khairul A Mastor, Neil McLatchie, Lindsay M Novak, Blessing N Onyekachi, Muhammad Rizwan, Mark Schaller, Eleonora Serafimovska, Eunkook M Suh, William B Swann, Eddie M W Tong, Ana Torres, Rhiannon N Turner, Christin-Melanie Vauclair, Alexander Vinogradov, Zhechen Wang, Victoria Wai Lan Yeung, Brock Bastian

**Affiliations:** Melbourne School of Psychological Sciences, University of Melbourne, Melbourne, Victoria, 3052, Australia; School of Psychology, University of Queensland, Brisbane, Queensland, 4072, Australia; Department of Experimental and Applied Psychology, Vrije Universiteit Amsterdam, Amsterdam, 1075 BT, The Netherlands; Department of Psychology, University of Southern California, Los Angeles, CA 90089, USA; Melbourne School of Psychological Sciences, University of Melbourne, Melbourne, Victoria, 3052, Australia; Department of Psychology, Université du Québec à Montréal, Montreal, Quebec, H2L 2C4, Canada; Institute of Psychology, University of Tartu, Tartu, 50090, Estonia; Department of Educational, Organizational and Social Psychology, Makerere University, Kampala, Uganda; Department of Psychology, Cheikh Anta Diop University, Dakar, 10700, Senegal; CLLE, CNRS, Université de Toulouse, Toulouse, 31058, France; Faculty of Psychology, University of Warsaw, Warsaw, 00-183, Poland; Faculty of Psychology, Chulalongkorn University, Bangkok, 10330, Thailand; Faculty of Arts and Social Sciences, Sabanci University, Istanbul, 34956, Turkey; Serra Húnter Fellow, Department of Psychology, University of Girona, Girona, 17004, Spain; Department of Psychology, University of Milano-Bicocca, Milan, 20126, Italy; Department of Psychology, University of Latvia, Riga, LV-1586, Latvia; Departamento Académico de Psicología, Pontificia Universidad Católica del Perú, Lima, 15088, Peru; Psychology Department, University of the Witwatersrand, Johannesburg, 2017, South Africa; School of Psychology, Victoria University of Wellington, Wellington, 6012, New Zealand; Department of Psychology, Saarland University, Saarbrücken, 66123, Germany; Facultad de Psicología, Universidad Nacional de Educación a Distancia, UNED, Madrid, 28040, Spain; Escuela de Psicología, Pontificia Universidad Católica de Chile, Santiago, 8331150, Chile; Graduate School of Social Sciences, Hitotsubashi University, Kunitachi, 186-8601, Japan; Centre of Social and Psychological Sciences, The Slovak Academy of Sciences, Bratislava, 814 38, Slovakia; School of Psychological and Behavioral Sciences, Southern Illinois University, Carbondale, IL 62901, USA; Department of Psychology, University of San Carlos, Cebu City, 6000, Philippines; School of Psychology, Swansea University, Swansea, Wales, SA2 8PP, UK; Faculty of Psychology and Educational Sciences, KU Leuven, Leuven, 3000, Belgium; School of Philosophy, Psychology and Language Sciences, The University of Edinburgh, Edinburgh, Scotland, UK; School of Liberal Studies, Universiti Kebangsaan Malaysia, Bangi, 43600, Malaysia; Faculty of Science and Technology, Lancaster University, Lancaster, England, LA1 4YW, UK; Department of Psychology, University of Illinois Chicago, Chicago, IL, 60607, USA; Department of Psychology, University of Nigeria, Nsukka, 410105, Nigeria; Department of Clinical Psychology, National University of Medical Sciences, Rawalpindi, 46220, Pakistan; Department of Psychology, University of British Columbia, Vancouver, British Columbia, V6T 1Z4, Canada; Institute for Sociological Political and Juridical Research, Ss Cyril and Methodius University in Skopje, Skopje, 1000, Macedonia; Department of Psychology, Yonsei University, Seoul, 03722, South Korea; Psychology Department, The University of Texas, Austin, TX, 78712, USA; Department of Psychology, National University of Singapore, 119077, Singapore; Departamento de Psicologia, Federal University of Paraíba, João Pessoa, 58051-900, Brazil; School of Psychology, Queens University Belfast, Belfast, Northern Ireland, BT7 1NN, UK; Department of Social and Organizational Psychology, Instituto Universitário de Lisboa (ISCTE-IUL), CIS-IUL, Lisbon, 1649-026, Portugal; Faculty of Psychology, Taras Shevchenko National University of Kyiv, Kyiv, 01033, Ukraine; School of Social Development and Public Policy, Fudan University, Shanghai, 200433, China; School of Psychology, University of Queensland, Brisbane, Queensland, 4072, Australia; Department of Psychology, Lingnan University, Tuen Mun, Hong Kong, China; Wofoo Joseph Lee Consulting and Counselling Psychology Research Centre, Lingnan University, Tuen Mun, Hong Kong, China; Melbourne School of Psychological Sciences, University of Melbourne, Melbourne, Victoria, 3052, Australia

**Keywords:** moralization, economic inequality, anomie, moral judgments, Twitter

## Abstract

Throughout the 21st century, economic inequality is predicted to increase as we face new challenges, from changes in the technological landscape to the growing climate crisis. It is crucial we understand how these changes in inequality may affect how people think and behave. We propose that economic inequality threatens the social fabric of society, in turn increasing moralization—that is, the greater tendency to employ or emphasize morality in everyday life—as an attempt to restore order and control. Using longitudinal data from X, formerly known as Twitter, our first study demonstrates that high economic inequality is associated with greater use of moral language online (e.g. the use of words such as “disgust”, “hurt”, and “respect’). Study 2 then examined data from 41 regions around the world, generally showing that higher inequality has a small association with harsher moral judgments of people's everyday actions. Together these findings demonstrate that economic inequality is linked to the tendency to see the world through a moral lens.

**Significance Statement**

High economic inequality leads to a myriad of negative social and political outcomes, and evidence is beginning to emerge suggesting it may also affect how people engage with morality. Beliefs about right and wrong are a powerful determinant of behavior, the treatment of others, and tolerance for a pluralistic society. Understanding the role of economic inequality in shaping an approach to morality thus has a range of implications for the functioning of society more broadly. Using diverse methods, participant pools and outcomes, we demonstrate that high inequality is linked to a greater perception that the social and moral fabric of society is crumbling, and in turn, a greater tendency to emphasize morality in daily life.

## Introduction

In 2023, the World Economic Forum published an article sounding the alarm on rising economic inequality in the 21st century (1). The COVID-19 pandemic, the cost-of-living crisis, automation, and growing climatic disasters are predicted to compound in the coming decades to further intensify this wealth disparity (2, 3). In light of this trend, it is crucial to understand how economic inequality may affect interactions between people in society (4). We posit that economic inequality may enhance moralization—that is, the greater tendency to employ or emphasize morality in everyday life. This is because inequality erodes the social fabric of society (4–7). In response to perceived threats to control, individuals often compensate by engaging in behaviors that restore a sense of order (4, 7–13). We propose that enhanced moralization may be employed as a strategy—whether effective or not—to attempt to restore order and control. Here, we aim to find empirical evidence for the relationship between economic inequality—both the actual level of inequality in society as well as subjective perceptions of how unequal society is—and moralization. We measure moralization via the increased use of moral language in daily communication and harsher moral judgements about the actions of others.

Economic inequality not only creates a competitive economic climate but can also erode social cohesion in society (4). Inequality is known to cause fractured social connections, lower trust, reduced cooperation (5, 6, 14, 15), and increased competitive sentiments (16–18). Importantly, the perception of social fragmentation caused by economic inequality affects most individuals largely independent of their socioeconomic status (19). Indeed, high inequality leads to views that society is descending into a state of *anomie* (4, 5, 7, 12). Anomie is described as the view that society has become disintegrated (breakdown in the social fabric) and dysregulated (breakdown in leadership; 20, 21). The breakdown of the *social fabric* in society in particular has the potential to enhance moralization.

As an inherently cooperative species, humans are highly attuned to threats to the social order. When the social structure of society is crumbling, people begin to feel uncertain and act in ways to restore a sense of order and control. Sprong et al. (7) found that high inequality enhanced a sense of anomie and, in turn, increased a desire for a strong leader who would do what it takes to retake control over society. Likewise, high inequality, via increased perceptions of anomie, enhances conspiratorial thinking (12). The authors proposed that inequality undermines individuals' perceived control, making conspiracy theories more appealing because they offer a simpler framework for understanding complex social events. Together the literature suggests that economic inequality threatens the social fabric of society and individuals are motivated to restore a sense of order in response to this threat. This conclusion is also in line with classic theorizing which emphasizes that a loss of prediction and control can affect mental activity and social behavior, e.g. balance theory (22) and interdependence theory (23). Likewise, individuals often engage in defensive reactions such as protest when needs are thwarted, e.g. reactance as means to regain control (24).

We propose that individuals may also attempt to restore a sense of order and control by engaging in moralization. That is, when issues become a matter of right and wrong, this provides a high-certainty guide for how an individual should behave across contexts. Critically, morals not only provide a blueprint for how an individual should act but are also often standards that are imposed upon others. As such, moralization may be employed as an attempt to restore a sense of order in both the self and society more broadly.

Moralization is an umbrella term and can manifest in many ways. First, the tendency to view the world through a moral lens may be expressed through the use of moral language in public forums. Communicating moral standards in public settings may aid in clarifying how both the individual and broader community ought to think and behave, in turn establishing a sense of order and control. Second, enhanced moralization may be expressed through the harsh judgment of others. Harshly judging the actions of others reflects a firm commitment to one's personal moral code, in turn strengthening a sense of order and consistency in one's own conduct. Judging apparent misdeeds may also aid in restoring a sense of order and control in society more broadly, signaling that certain actions or opinions will not be tolerated and potentially serving as a precursor for punishment.

At first glance, this explanation—that high inequality erodes the social fabric and, in turn, increases moralization—may seem at odds with findings in the literature. While studies have linked high inequality to a range of outcomes deemed normatively “immoral”, such as escalated criminal activity (19, 25) and a decline in cooperation (14, 26–29), these behaviors should not be conflated with moralization itself. Indeed, moralization involves the increased propensity to view and interpret behaviors and situations through a moral framework, a lens that adds moral significance to a broader spectrum of social interactions. However, the relationship between moralization as a cognitive process and the manifestation of normatively moral or immoral actions is underexplored. Indeed, moralization is linked with a number of morally questionable outcomes, including the exclusion of others with dissimilar beliefs (30–32) and the endorsement of violence to achieve a moral end (33).

Economic inequality likely has a very nuanced effect on moral actions and moralization. On one hand, it can erode the foundational trust and mutual reliance necessary for cooperation, potentially leading to a rise in self-protective and cheating behaviors as individuals compete for scarce resources. On the other hand, the same conditions of inequality can prompt a heightened emphasis on moralization. As traditional social contracts are perceived to fail, some individuals may turn to moralization as a way to reassert order and values within a context that seems increasingly out of control. This explanation instead reflects a more nuanced picture where different responses emerge from individuals or groups within society when confronted with the stressors associated with economic inequality.

Past research hints that inequality may be linked to *greater* moralization; perceived threat to the social order of society predicts harsher punishment of criminals (11, 13, 34, 35). However, this work looks at the extreme end of immoral behavior (i.e. actions that are punishable by law) but cannot speak to whether people may enhance the use of moralization in day-to-day interactions. Other research has shown that various forms of threat—such as COVID-19 and social ostracism—are linked to harsher moral condemnation (36–38). However, it is unclear from this work whether economic inequality, a persistent and pervasive threat to social order, elicits a similar moral response, and whether this response also generalizes to language use in everyday contexts.

More recently, Elbæk et al. (39) found a direct link between high economic inequality in society and a number of self-report measures linked to moral virtue. That is, high inequality across 67 countries was related to viewing cooperation as a virtue (40) and the centrality of virtue to an individuals' self-concept (41). Yet, it is still unknown whether inequality may relate to an increased tendency to form moral attitudes and employ moral judgements in everyday life—through both the public declaration of moral stances as well as harsh judgments of the actions of others—and whether this can be explained by perceived threats to the social structure.

The current work aims to establish the relationship between economic inequality and everyday moralization across two studies with complementary methods. In particular, we examined whether inequality was linked to two manifestations of moralization that would aid in regaining a sense of order and control, via: (i) the use of moral language and (ii) the harshness of moral judgments. Our first study examined the link between inequality and moral language in a context where morals are frequently expressed: X (hereafter referred to by its previous name, Twitter). To achieve this, we assessed the link between inequality in towns across the United States and the use of moral language in Tweets over a period of 9 years. Across 41 locations around the world, Study 2 then explored how both objective indicators and subjective perceptions of inequality relate to perceptions that the social fabric is crumbling and, in turn, the harshness of moral judgments. Together, these studies aim to shed light on how and why economic inequality may be linked to increased moralization (42–44).

## Results

### Study 1—Moral language on Twitter

In the social media age, much of our moral dialogue occurs online. Platforms such as Twitter run on a business model where the goal is to maintain attention, resulting in a greater flow of negative and rage-inducing content (42–46). Unlike other platforms such as Facebook or Instagram, Twitter focuses more on worded content over images and shows people a significant portion of content outside their chosen network. Twitter is therefore a naturalistic environment geared towards the sharing of moral content to a wide network. Here, we aimed to assess whether inequality relates to the use of moral words in Tweets. Using a random sample of six billion Tweets, we assessed the number of moral words used in posts that were geolocated to a “place code” (e.g. city, town, or municipality) in the United States per year, from 2012 to 2020.

Moral Foundations Theory dictates that moral concern can be placed into six core subtypes—care/harm, fairness/cheating, liberty/oppression, authority/subversion, purity/degradation, and loyalty/betrayal (47, 48)—with individuals varying on the degree to which they adopt each “foundation”. Using the moral foundations dictionary (49), we assessed the number of moral words in Tweets overall, as well as the words that relate to five categories of moral concern: care/harm (e.g. “compassion” or “kill”), fairness/cheating (e.g. “equal” or “bigot”), authority/subversion (e.g. “respect” or “dissent”), purity/degradation (e.g. “innocent” or “disgust”), and loyalty/betrayal foundations (e.g. “solidarity” or “deceive”; 35–37)^1^. We additionally assessed the use of *virtue* (e.g. “help”) and *vice* (e.g. “hurt”) words within each of these categories. Finally, we examined *individualizing* words as a sum of care/harm and fairness/cheating words (i.e. those related to treatment of individual beings—typically adopted across the political spectrum), as well as *binding* words as a sum of authority/subversion, purity/degradation, and loyalty/betrayal words (i.e. those related a group's wellbeing or cohesion—typically adopted by political conservatives; 35, 37).

We examined the role of economic inequality on the number of moral words using objective Gini indices from each city in the United States per year. The Gini index is a common indicator of how un/equal wealth is spread across a certain population, with higher values indicating greater inequality. Information on the calculation of the Gini coefficient can be found in Supplementary Materials. Religiosity, presidential voting behavior, and gross domestic product (GDP) were additionally sourced to include as control variables. We hypothesized that greater inequality would predict more moral language used in Tweets.

The full results for all models reported below can be seen in Supplementary Materials. We conducted ANOVAs to compare the Akaike Information Criterion (AICs) of models with various random effect structures to establish which was most optimal. Based on these results, we included (i) year and (ii) city nested within county nested within state, as random intercepts in all models reported below. Ten negative binomial generalized linear mixed models (GLMM) were used to assess the relationship between the Gini index and (i) moral words more generally, (ii) vice and virtue words, (iii) individualizing and binding foundations, and (iv) for each of the five foundations specifically. Table 1 presents summary results for each of these models using unstandardized indices. Higher inequality was associated with the greater use of moral words (total), vice and virtue words, individualizing and binding words, and each of the five foundations individually. Incidence rate ratios (IRRs) suggest that a 0.1 unit increase in the Gini coefficient corresponds to a 6.5% increase in the total number of moral words used. We assessed the robustness of our key finding—that high inequality relates to the use of moral words (total)—by including the Gini index as a random slope for both year and place code^2^, and results remained consistent, *b* = 1.08, SE = 0.13, *P* < 0.001. Economic inequality remained a significant predictor for the remaining nine ways of categorizing the moral words (vice, virtue, individualizing, harm, fairness, binding, purity, authority, and loyalty) when the Gini index was included as a random slope (see Supplementary Materials for results).

**Table 1.** Unstandardized indices for negative binomial generalized linear mixed models examining the effect of Gini Index on the use of moral words in tweets.

Outcome variable	*b*	SE	*P*	IRR^0.1^
Moral words (overall)	0.63	0.08	<0.001***	1.065
Vice	0.91	0.09	<0.001***	1.095
Virtue	0.56	0.08	<0.001***	1.058
Individualizing	0.69	0.08	<0.001***	1.071
Harm	0.70	0.08	<0.001***	1.072
Fairness	1.29	0.11	<0.001***	1.137
Binding	0.74	0.08	<0.001***	1.077
Purity	0.96	0.10	<0.001***	1.100
Authority	1.20	0.11	<0.001***	1.128
Loyalty	1.00	0.10	<0.001***	1.105

IRR, incidence rate ratio. Original IRR values have been adjusted and the values given can be interpreted as the change in moral word usage associated with a 0.1 increase in the Gini coefficient.

*P < 0.05. **P < 0.01. ***P < 0.001.

We assessed whether our results for the total number of moral words held when controlling for other variables, including GDP, religiosity, and voting behavior. Our significant finding for the total moral words score held when controlling for these variables, such that higher inequality predicted more moral words in Tweets, *b* = 0.61, SE = 0.07, *P* < 0.001. Results also remained significant for all nine ways of categorizing the moral words (vice, virtue, individualizing, harm, fairness, binding, purity, authority, and loyalty) when controlling for GDP, religiosity, and voting behavior (see Supplementary Materials). We then lagged our data by 1 year to assess whether Gini index at time 1 predicted the use of moral words (overall) in Tweets at time 2, controlling for Gini at time 2 and moral words (overall) in Tweets at time 1. We divided the moral words control variable (i.e. at time 1) by the total number of Tweets to adjust for areas with greater volumes of posts as our offsetting function only affects the dependent variable. Results demonstrated that a higher Gini coefficient at time 1 (the prior year) significantly predicted a greater use of moral words in tweets at time 2 (the following year), *b* = 0.61, SE = 0.11, *P* < 0.001. In the same model, Gini at time 2 was still related to moral word use at time 2 (i.e. measurements at the same time point), albeit greatly reduced, *b* = 0.32, SE = 0.10, *P* = 0.002. This indicates that while current economic inequality still is related to moral word usage, its effect is less pronounced compared to the influence of historic economic conditions.

In Study 1, we found clear evidence that greater inequality predicted the use of moral words in Tweets in the United States, across a period of 9 years. This relationship was replicated when looking at moral words generally, the use of vice and virtue words, individualizing and binding foundations, and each of the five foundations separately. We further found evidence that hints at a causal pathway—namely, that high inequality predicted more moral words used 1 year later. While this work provides evidence for the link between inequality and more moral language use online, this is a crude level of analysis that only hints at enhanced engagement with morality in daily life. A critical expression of moralization is imposing one's moral framework onto the actions of others. In Study 2, we turned to the relationship between economic inequality and the harshness of moral judgments about the behavior of others in a multinational sample.

### Study 2—Moral judgments across 41 cultures

For our second study, we aimed to establish the link between high economic inequality and the tendency to make harsher moral judgments about actions across 41 locations around the world. We assessed economic inequality on both the country-level (objective indicator, via online indices from the World Bank) as well as subjective perceptions of inequality. Many individuals may not know how unequal their society actually is (50) and individuals in the same country may experience more equal or unequal local environments (51). Subjective and objective inequality only moderately correlate (5, 52) and prior work has shown that subjective perceptions of wealth are often more predictive of psychological outcomes relative to objective measures (5, 7). Perceptions of inequality were measured by asking participants to estimate the proportion of individuals who they believe fit into different wealth categories (i.e. very poor, poor, average in wealth, wealthy and very wealthy). These numbers were then used to calculate a perceived Gini coefficient (see Supplementary Materials for calculation).

To explore moral judgments, we used the Clifford vignettes (47) and asked participants to judge how wrong a variety of scenarios were that spanned the six domains of moral concern: harm, fairness, liberty, authority, loyalty, and purity. For example, participants were shown the following scenarios and asked to judge how wrong they are: “You see a boy placing a thumbtack sticking up on the chair of another student” (harm) and “You see a teenage girl coming home late and ignoring her parents’ strict curfew” (authority). We examined the tendency to make harsher judgments collapsed across all scenarios, as well as the individualizing (harm, fairness, and liberty) and binding (authority, loyalty, and purity) scenarios. Finally, we assessed the harshness of moral judgments for each foundation specifically. Critically, we tested whether perceptions of anomie in the social fabric of society explained the relationship between high economic inequality and harsher moral judgments. We controlled for economic and social conservativism, gender, age, subjective social status, religiosity, and GDP at purchasing power parity (GDP PPP) in all analyses. We hypothesized that higher inequality, both perceived and objective, will be linked to the general tendency to make harsher moral judgments about others' actions.

**Fig. 1.**
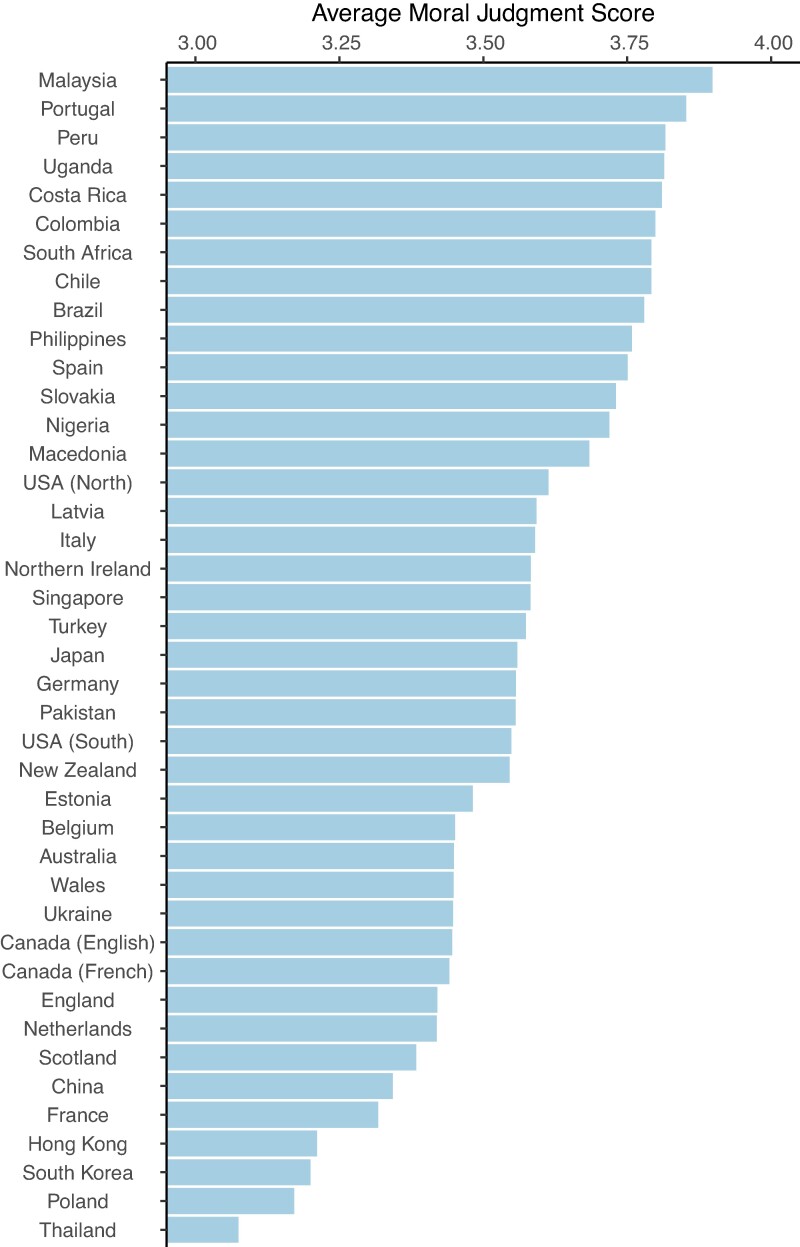
Average moral judgment score across locations. Higher values indicate harsher moral judgments.

We ran Pearson's correlations to assess the relationship between our two inequality indicators and subjective social status. The perceived Gini coefficient had a small to moderate correlation with the objective Gini coefficient (*r* = 0.35, *P* < 0.001). However, subjective status only had a small correlation with both the perceived (*r* = −0.05, *P* < 0.001) and the objective (*r* = −0.03, *P* = 0.046) Gini coefficients. See Supplementary Materials for full results for all models reported below. Based on the intraclass correlation, approximately 13.4% of the variance in moral judgments can be explained at the country level^3^ (see Fig. 1; see Supplementary Materials for average country score per foundation). Likewise, approximately 16.3% and 19.5% of the variance in the individualizing and binding foundations, respectively, can be explained by differences between countries. To establish the relationship between moral judgments and the control variables, an LMM was conducted. As shown in Table 2, females (*M* = 3.59, SD = 0.52) tended to make slightly harsher moral judgments relative to males (*M* = 3.51, SD = 0.56). Moral judgments also became harsher with age, and participants tended to more harshly judge the moral scenarios when they had a lower subjective social status. Harsher moral judgments were also witnessed with both increased importance of religion and social conservatism (relative to social liberalism).

**Table 2.** Linear mixed model examining the effect of control variables on moral judgments .

Predictors	*β*	95% CI	*P*
GDP PPP per capita	−0.11	−0.23 – 0.00	0.055
Gender (female)	0.29	0.24–0.34	<0.001***
Age	0.04	0.01–0.07	0.005**
Subjective social status	−0.03	−0.06 – −0.01	0.008**
Social conservativism	0.09	0.06–0.12	<0.001***
Economic conservativism	−0.03	−0.06–0.00	0.063
Importance of religion	0.12	0.10–0.15	<0.001***
**Random effects**			
Residual	0.82		
Country (intercept)	0.12		
ICC	0.13		
Observations	6,019		
Marginal *R*^2^/Conditional *R*^2^	0.063/0.180		

Gender was coded as male (1) and female (2). Marginal *R*^2^ refers to fixed effects only and conditional *R*^2^ refers to the entire model. Standardized beta values provided.

*P < 0.05. **P < 0.01. ***P < 0.001.

**Fig. 2.**
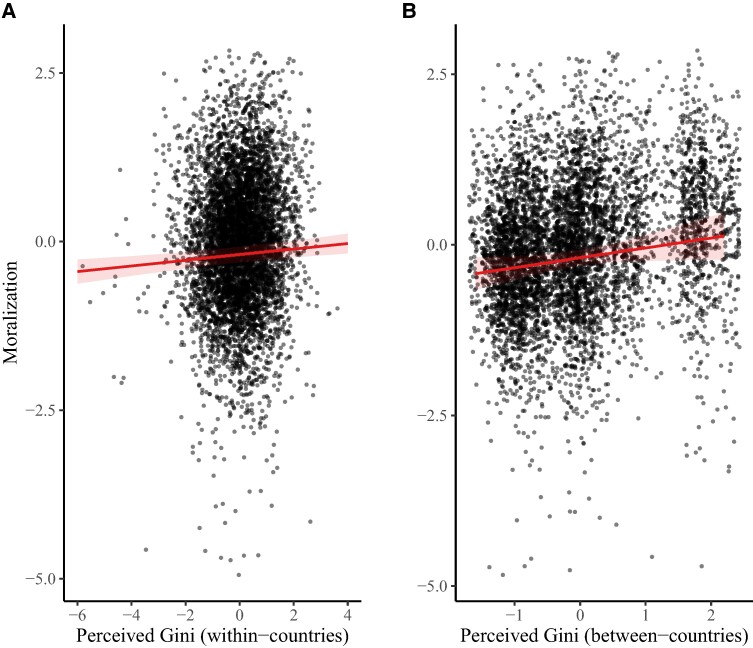
The relationship between the perceived Gini coefficient and moral judgments within-countries (A) and between-countries (B). All variables have been scaled and centered. Each point has been jittered for ease of interpretation and the light red area surrounding the trend line represents confidence intervals.

**Fig. 3.**
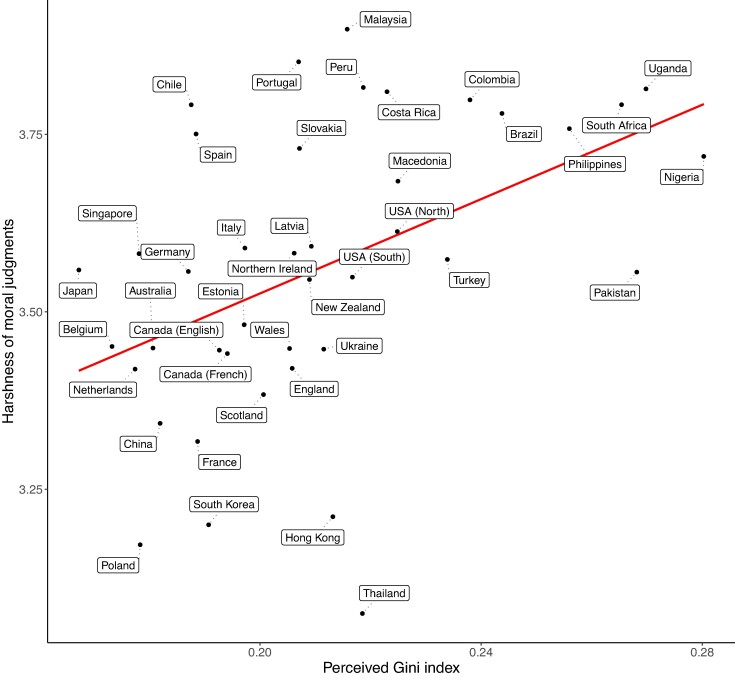
The relationship between the average perceived Gini index and moral judgment score by country.

All control variables were included in each of the models reported below. We conducted an LMM to examine the effect of country-level objective Gini on moral judgments. Results revealed a larger objective Gini index (i.e. more economic inequality) was associated with harsher moral judgments overall, *β* = 0.20, SE = 0.06, *P* = 0.003. A further LMM revealed that a larger perceived Gini index (i.e. greater inequality) was associated with harsher moral judgments overall within-countries, *β* = 0.04, SE = 0.01, *P* < 0.001, but this relationship was only on the cusp of significance between-countries, *β* = 0.14, SE = 0.07, *P* = 0.051 (see Fig. 2). See Fig. 3 for the relationship between the average perceived Gini index and the harshness of moral judgments by country. We ran several exploratory LMMs to check the moral judgments effect is not being driven by any specific moral foundation. Table 3 demonstrates some mixed findings but indicates that there is general evidence that greater inequality is linked to harsher moral judgments across a variety of moral concerns. In general, the effects produced are small.

**Table 3.** Sixteen linear mixed models examining the effect of inequality predictors on moral foundation vignettes.

		Within-country effects			Between-country effects		
Predictor	Outcome	*β*	95% CI	*P*	*β*	95% CI	*P*
Perceived Gini	Individualizing	0.03	0.01–0.05	0.007**	−0.07	−0.24–0.10	0.415
	Harm	0.02	0.00–0.04	0.125	−0.11	−0.27–0.05	0.164
	Fairness	0.02	−0.01–0.04	0.188	0.02	−0.12–0.16	0.775
	Liberty	0.04	0.02–0.06	0.001**	−0.08	−0.26–0.10	0.367
	Binding	0.03	0.01–0.06	0.002**	0.26	0.13–0.39	<0.001***
	Purity	0.02	−0.01–0.04	0.186	0.22	0.08–0.36	0.003**
	Authority	0.02	−0.00–0.04	0.090	0.19	0.06–0.32	0.006**
	Loyalty	0.05	0.02–0.07	<0.001***	0.20	0.10–0.31	<0.001***
Objective Gini	Individualizing	—	—	—	0.17	0.02–0.32	0.026*
	Harm	—	—	—	0.06	−0.09–0.22	0.404
	Fairness	—	—	—	0.19	0.07–0.30	0.002**
	Liberty	—	—	—	0.15	−0.01–0.32	0.063
	Binding	—	—	—	0.16	0.02–0.30	0.024*
	Purity	—	—	—	0.11	−0.04–0.25	0.138
	Authority	—	—	—	0.18	0.06–0.30	0.005**
	Loyalty	—	—	—	0.11	−0.01–0.22	0.063

Each line denotes a separate linear mixed model, where both the within-country and between-country effects are included in the same model. Standardized beta values provided.

*P < 0.05. **P < 0.01. ***P < 0.001.

We then tested whether perceptions of anomie in the social fabric of society was related to economic inequality and moral judgments. High economic inequality was correlated with greater perceptions of anomie in the social fabric, for both the objective Gini index (*r* = 0.21, *P* < 0.001) and perceived Gini measure (*r* = 0.20, *P* < 0.001). Likewise, greater perceptions of anomie were correlated with harsher moral judgments in general (*r* = 0.16, *P* < 0.001), as well as harsher judgments for individualizing (*r* = 0.13, *P* < 0.001) and binding (*r* = 0.13, *P* < 0.001) foundations (see Supplementary Materials for correlations with specific foundations).

**Fig. 4.**
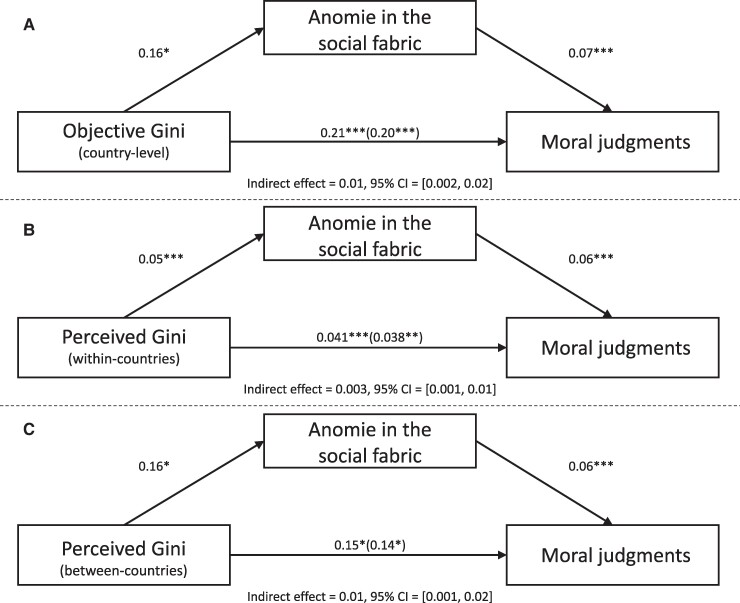
Effect of economic inequality measures on moral judgments, via anomie in the social fabric. *Note.* The three mediation analyses represent the link between the objective Gini (Panel A), perceived Gini (within-countries; Panel B), and perceived Gini (between-countries; Panel C) coefficients on moral judgments via anomie in the social fabric. Indirect effects were calculated for each of 1,000bootstrapped samples, with the 95% confidence intervals calculated for the 2.5th and 97.5th percentiles. The value outside parentheses on the lower path is the total effect, and the direct effect is the value inside parentheses. CI, confidence interval.

We analyzed whether anomie in the social fabric mediated the relationship between economic inequality and moral judgements in a multilevel mediation model. The indirect effect of objective Gini index via anomie in the social fabric on harshness of moral judgments (total) was significant (see Fig. 4A). Anomie in the social fabric also mediated the relationship between perceived Gini index (within- and between-countries) and harsh moral judgments (see Fig. 4B and C). The significant indirect effect was replicated for moral judgments for individualizing and binding foundations, as well as almost every moral foundation (see OSF and R script for all mediations). However, we note that each of the indirect effects for the total moral judgment scores were on the cusp of significance.

We found that higher inequality was linked to harsher moral judgments more generally, and this effect was consistent when examining both objective inequality on the country-level as well as individual perceptions of how unequal society was. The fact that this relationship also occurred on the country-level discounts alternative individual-level explanations, such as those who make harsher moral judgments are also more attuned to unfairness, and thus inequality, in their environment. Results were mixed when examining the effect of inequality on each foundation more specifically, with high perceived inequality linked to individualizing and binding foundations as well as liberty and loyalty, within-countries, and linked to all three binding foundations, between-countries. Likewise, high objective inequality on the country-level was linked to harsher moral judgments in both individualizing and binding foundations, but this effect may have been driven more specifically by judgments about violations of fairness and authority. Mediation analyses further revealed some evidence that the link between high inequality (for both objective and subjective Gini measures) and the harshness of moral judgments may be explained via enhanced perceptions of anomie in the social fabric of society. Indirect effects were found for moral judgments in general, individualizing and binding foundations, and in almost every case for the specific moral foundations, with the exception of loyalty (see Supplementary Materials).

Despite some variation in findings, these results suggest that high inequality is not only linked to either individualizing or binding foundations, nor is it only linked to judgments that are more closely related to inequality (e.g. fairness concerns). Rather, high inequality seems to be related to a general tendency to make harsher moral judgments. The results from Study 2 demonstrate that high inequality is linked to harsher moral judgments about the everyday actions of others, suggesting individuals are more attuned to and critical of how others behave.

## Discussion

High economic inequality is predicted to increase in the future as we grapple with major societal changes in the 21st century (2, 3), and it is critical to understand how these changes may affect how individuals think and behave (4). Across two studies, we found that high economic inequality is linked to a greater tendency to engage in moralization. Our first study found that higher inequality was related to the greater use of moral language on Twitter. Places in the United States with higher inequality were associated with more moral words in Tweets, and higher inequality at a previous timepoint was linked to more moral words in Tweets at a later timepoint. Spanning 41 locations around the world, Study 2 found that both objective, country-level inequality, and subjective perceptions of inequality had a small relationship with harsher judgments of everyday behaviors across a broad range of moral categories. Critically, the link between high inequality and harsher moral judgments was explained by greater perceptions of anomie in the social fabric of society. However, future work should replicate these findings as the indirect effects were small and on the cusp of significance. Combined, these diverse methods and participant pools suggest higher inequality is linked to greater moralization, potentially in response to the deteriorating social structure.

High economic inequality is known to erode the social fabric of society, demonstrated in both past work (4–7) and the findings presented here. As an inherently group-based species, cooperative networks are imperative for survival, and people may be particularly attuned to threats to social order. Prior research suggests that people may act in ways to regain a sense of order and control when facing environmental threats such as economic inequality (4, 8, 9, 11, 13, 34, 53). The current series of studies demonstrate that we may moralize more when exposed to high inequality, as a response to the degradation of social order in society. However, experimental evidence is needed in future work to confirm causality.

Our findings demonstrate that people may attempt to restore a sense of order and control by engaging in moralization via increasing the use of moral language and harshly condemning the misdeeds of others. Critically, inequality was not just linked to the tendency to consider moral matters closely related to unequal distributions (e.g. concerns related to fairness), nor is it only linked to moral concerns that are traditionally adopted by only one side of the political spectrum. Rather, inequality appears to relate to an increased tendency to see the world through a moral lens in a very general way, extending across a broad range of moral content.

It is important to note that while we have found the first evidence suggesting moralization may be employed by individuals as an *attempt* to restore a sense of order and control, it was not our goal to establish whether this approach to moral issues *actually* results in this outcome. Though evidence indicates that strong moral beliefs may bind individuals into groups (20, 48, 54), the same convictions can lead to the splintering of society, particularly in diverse and pluralistic environments. As moralization intensifies, this can lead individuals and groups to entrench themselves in moral positions—views which are typically less open to compromise compared to nonmoral beliefs (55). Indeed, high inequality is linked to greater polarization in society (56, 57), and research also suggests that moralization leads to polarizing outcomes, such as greater distancing from those with dissimilar beliefs (30–32) and increased partisan bias (30). Future work should focus on when, why, and how moralization may (or may not) be an effective way of restoring a sense of order and control, for both the individual and society more broadly.

It is also worth considering an alternative explanation for the function of moralization. Henderson and Schnall (58) propose a threat-monitoring framework, where they argue that when coping resources are low, being cautious of wrongdoers helps mitigate against the likelihood of additional threats to the self. Indeed, research suggests that those who face various threats to the self (e.g. concern about catching COVID-19 and social ostracism) make harsher moral judgments in general (36, 37). This framework can be feasibly extended to the current findings; economic inequality creates a threatening social environment (4, 5, 7), and individuals may be highly attuned to the threat of additional transgressions. This self-protective response differs from the mechanism we propose, which focuses on how individuals strive to maintain order and control in response to threats, both internally (i.e. providing a blueprint for one's own behavior) and externally (i.e. providing standards for behavior in society more broadly). In future, research should focus on confirming the function (or functions) of moralization in response to threat.

Our results are additive to an emerging body of literature, demonstrating a link between high economic inequality and approaches to morality (39). Elbæk et al. assessed a variety of self-report measures across 67 countries, finding a link between high inequality and the greater internalization of a broad range of moral virtues. Until now, however, it was not known whether inequality relates to the moralization of everyday attitudes and behaviors, and therefore whether morality is more relied upon in social communication and the judgment of others' everyday actions. If moralization is used as an attempt to restore a sense of order and control, it is crucial to assess outcomes that reflect how morality is used within everyday contexts to establish clear standards and rules for the self and others. Future work may wish to build upon our findings and examine how high inequality relates to other methods of restoring a sense of order, such as engaging in social punishment (e.g. via gossip, ostracism, or direct confrontation).

The effects observed in these studies are important when considering the ever-increasing use of social media to communicate with the broader community. Many of these platforms are designed (whether directly or indirectly) to amplify and spread moral outrage (42, 45). If inequality amplifies moralization, these effects may be even further compounded by the use of social media. Many have raised concerns about the negative effects of moral outrage on social media for building a trusting, cohesive, and forgiving society (46). It is therefore critical future work better understands the role of social media is perpetuating the effects of moralization in response to economic inequality.

The current work is a critical step in our understanding of the socio-environmental factors that enhance moralization. Using both correlational and longitudinal methods, we found evidence for the link between high inequality and moralization in both real-world discourse and hypothetical moral judgments. Critically, our approach combats the W.E.I.R.D. (Western, Educated, Industrialized, Rich, Democratic) bias frequently found in psychological research (59), validating our findings across nations and in a representative sample of social media users.

The current study also has raised several directions for future work. Study 1 only provided a broad analysis of moral content in Tweets. It remains unclear how people are using moral words in their Tweets, for example whether individuals are outraged, judging the misdeeds of others, or engaging in a more open discussion of moral issues. Moreover, while Study 2 included individuals from many nations around the world, the participants exclusively came from university samples. Future work should replicate our findings with more representative populations. Finally, morality can be examined from many other angles (e.g. attitudes on specific issues, virtue-signaling, rule-based or consequentialist approaches), and future research should uncover which manifestations of morality may be affected by economic inequality.

Every day we look at the world through a moral lens—we aim to do the right thing, and we judge others when they have committed a wrong. Our morals are a powerful determinant of our behavior and how we treat the people around us, yet we know little about how the structure of society can affect a tendency to moralize. Across 41 culturally diverse locations and online on Twitter, we found that high economic inequality is linked to harsher moral judgments and the increased use of moral language, respectively. Critically, our evidence shows that this effect occurs across a broad range of moral content and is not specific to any one issue. Combined, these results suggest that economic inequality may enhance the tendency to moralize in response to the deteriorating social fabric of society. As we navigate the complexities of the 21st century, it is critical we understand the influence of societal structures on our moral perspectives, and how we treat others.

## Materials and methods

### Study 1—Moral language on Twitter

Ethical approval was obtained by the fourth author from the University of Melbourne (application ID: 26005).

#### Procedure

To extract moral posts on Twitter, we used a previously validated dictionary of moral words—the moral foundations dictionary (49). We further validated the use of each term by searching Twitter and confirming that each word was typically used in a moral context (see Supplementary Materials for amended dictionary). The dictionary was subdivided into 11 categories: general moral words, as well as a virtue (moral words that would be typically classified as positive e.g. “help’) and vice (moral words that would be typically classified as negative e.g. “hurt’) category for five moral foundations: harm, fairness, authority, loyalty, and purity.

We applied this dictionary of words to a database of 6 billion Tweets spanning the years 2012 to 2020. This pool of Tweets was downloaded from the Sprinkler Application Programming Interface (API), which provides a random sample of approximately 1% of the public Twitter feed. Each Tweet was geolocated to a “place code” (e.g. city, town or municipality) in the United States using a previously validated geolocation algorithm (60), resulting in a total of 5,434 cities per year. We ascertained the number of Tweets that contained at least one word for each of the 11 moral categories as well as the total number of Tweets more generally from that location to control for places with greater Tweet volume. Approximately 28 million Tweets contained at least one moral word.

#### Materials

To model the effect of economic inequality on Tweets with moral words, we gathered the Gini indices for each city and year from the US Census Bureau (61). The Gini index ranged from 0 (least inequality) to 1 (most inequality). We also gathered several control variables to assess the robustness of our findings. First, we used real Gross Domestic Product (GDP)—a measure that has been adjusted for inflation—per year on the county-level from the Bureau of Economic Analysis (62), as income inequality can be associated with economic growth (63), and prior work has shown that scarcity of resources predicts a stronger moral identity (39). We further controlled for religiosity, as places with higher inequality are typically more religious (64), and religious individuals tend to adopt group binding principles more so than nonreligious individuals (65). The data for religiosity were obtained from the Pew Research Centre (66) with only one time point available on the state level and dictate the percentage of people who believe that religion was “very important” to them. Finally, we controlled for political orientation as there are significant differences between political liberals and conservatives in the moral foundations they typically adopt (48), and liberals perceive greater levels of inequality relative to conservatives (67). We thus included presidential election data and more specifically, the percentage of individuals who voted for the Republican party (68). This data was available on the 4-year election cycle for each county, and we thus applied the vote percentage to the proceeding 4 years after an election (i.e. 2012 results applied for the years spanning 2012 to 2015).

#### Method of analysis

Given our dependent variable was count data (i.e. number of Tweets), we used negative binomial generalized linear mixed models (GLMM) to assess the effect of economic inequality on the number of moral words in Tweets. We accounted for areas that had larger Tweet volumes by offsetting the total number of Tweets more generally from each place, and included two random intercepts: (i) year and (ii) place nested within county nested within state. We first assessed the effect of economic inequality on (i) moral words more generally, (ii) vice and virtue words, (iii) individualizing and binding foundations, and (iv) for each of the five foundations specifically. We then tested the robustness of our results by assessing the effect of economic inequality on total number of moral words, controlling for GDP (scaled), religiosity and voting behavior. Finally, to better understand potential causality, we lagged our data and explored whether the Gini index at time 1 predicted moral word count at time 2, controlling for Gini at time 2, and moral word count at time 1.

### Study 2—Moral judgments across 41 cultures

Ethical approval was obtained by the last author from the University of Melbourne Behavioural and Social Sciences Ethical Review Committee (project no. 2009001486). Informed consent was obtained in line with the requirements of ethical approval. This study meets the relevant ethical guidelines for each country involved. This study drew on data from an existing multinational dataset and has been used for other studies with diverging hypotheses (5, 69, 70).

#### Participants

Participants were recruited between 2018 and 2019 from 41 universities across 35 countries: Australia, Belgium, Brazil, Canada (English speaking), Canada (French speaking), Chile, China, Colombia, Costa Rica, England, Estonia, France, Germany, Hong Kong (HKSAR, China), Italy, Japan, Latvia, Macedonia, Malaysia, Netherlands, New Zealand, Nigeria, Northern Ireland, Pakistan, Peru, Philippines, Poland, Portugal, Scotland, Singapore, Slovakia, South Africa, South Korea, Spain, Thailand, Turkey, Uganda, Ukraine, United States (North), United States (South), and Wales^4^. In total, 6,665 participants (*M* = 21.59 years, SD = 5.72 years; 63% female) completed the questionnaire. See Supplementary Materials for information regarding sample size and data collection.

#### Measures

The individual measures discussed below were taken from a larger multinational survey^5^, and country-level measures were taken from existing online databases. Details of the individual-level measures can be found in Supplementary Materials.

*Moral judgments.* We assessed how wrong participants believed various actions were through a selection of Clifford Vignettes (47) that detail a variety of potentially morally relevant scenarios spanning the six moral foundations. Participants were presented with 24 scenarios and were asked to judge how morally wrong they consider each of the behaviors on a scale from 1 (not at all wrong) to 5 (extremely wrong). Participants were asked nine harm items (*α* = 0.82), spanning physical harm towards humans (three items, e.g. “You see a woman spanking her child with a spatula for getting bad grades at school”), psychological harm towards humans (three items, e.g. “You see a girl laughing at another student for forgetting her lines in a school play”) and physical harm towards animals (three items, e.g. “You see a boy setting a series of traps to kill stray cats in his neighborhood”). Items also assessed fairness (three items, e.g. “You see a politician using federal tax dollars to build an extension on his home”; *α* = 0.65), liberty (three items, e.g. “You see a man forbidding his wife to wear clothing that he has not first approved”; *α* = 0.63), loyalty (three items, e.g. “You see a teacher publicly saying she hopes another school wins the math contest”; *α* = 0.73) and authority violations (three items, e.g. “You see an employee trying to undermine all of her boss' ideas in front of others”; *α* = 0.72). Two items were used to assess the purity foundation (e.g. “You see a man searching through the trash to find women's discarded underwear”)^6^. We created a total moral judgment measure by averaging the means of each foundation (*α* = 0.73). This approach adjusted for the higher number of harm items and lower number of purity items to ensure that each foundation was appropriately weighted in the combined measure. Using a similar approach, we also averaged the means of the harm, fairness, and liberty items to create an individualizing measure (*α* = 0.72), and averaged the means of the loyalty, authority, and purity foundations to create a binding measure (*α* = 0.70). While we took care to weight items appropriately, we note the limitations of having different number of items per foundation.

*Inequality.* We measured inequality in two ways: objective Gini index (country-level) and perceived Gini index (individual-level)^7^. We first included a measure of country-level, objective economic inequality with the Gini index from The World Bank (71). This assesses the degree to which wealth is un/evenly spread in a population.

We also measured subjective perceptions of inequality using a quasi-Gini index. Participants were told to imagine 100 members of their country and asked to dictate how many of these 100 people they thought were “very poor”, “poor”, “average in wealth”, “wealthy”, and “very wealthy”. See Supplementary Materials for information on the calculation of this measure. Both objective and perceived Gini measures were calculated in a similar way, and scores could range from 0 (most equal), to 1 (most unequal).

*Perceptions of anomie in the social fabric.* Six items were used to assess the breakdown in the social fabric of society, as reflected in the perceived degradation of cooperation, trust, and shared moral standards amongst citizens (21). All items were averaged, for example “People think that there are no clear moral standards to follow” and “People do not know who they can trust and rely on”. Responses were assessed on a scale from 1 (strongly disagree) to 7 (strongly agree), with higher scores indicating greater perceptions of anomie in the social fabric of society (*α* = 0.77).

*Control variables.* We controlled for several variables that may be related to moral judgments and levels of economic inequality. Liberals and conservatives tend to differ in their adoption of the moral foundations (47, 48), and liberals are more likely to believe inequality is greater compared to conservatives (67). To account for this, we measured economic and social conservatism, and responses to both questions were coded from 1 (left/liberal), to 7 (right/conservative). The adoption of certain foundations tend to differ by gender (48) and females also tend to perceive lower inequality relative to males (67). We thus measured gender as 1 (male) or 2 (female). Age was measured in years. Social status is known to affect perceptions of how wealth is distributed (51, 67), and we controlled for social status using the MacArthur Scale of Subjective Social Status (72–74). Participants were shown a 10-rung ladder and asked to indicate where they felt they fit on the ladder relative to others in their society in terms of money, education, and job prestige, and this was coded from 1 (bottom rung/worst off in society), to 10 (top rung/best off in society). This measure differs from the perceived Gini index as it assesses a personal evaluation of where one fits into society relative to others. On the other hand, the perceived Gini index focuses on perceptions of society more broadly and measures how un/equally economic resources are spread in a given population.

Certain foundations are also more likely to be adopted by those who are religious (e.g. purity; 37) and religious countries also tend to be more unequal (64). We accounted for this by including a measure of the importance of religion. Participants were asked if they followed a religion, and if so, how important religion is in their daily life. Responses were recorded from 1 (not at all important) to 7 (extremely important). For those who do not follow a religion, their missing data was recoded as 1. Finally, we accounted for the wealth of each country as economic growth can correlate with inequality (63). We thus included a measure of gross domestic product at purchasing power parity (GDP PPP) per capita from the World Bank in international dollars (75).

#### Method of analysis

Our data came from 41 samples, and this was accounted for by using a series of linear mixed models (LMM), with a random intercept of country. The analyses were conducted in R studio (76) with the lme4 package to estimate models (77). We included the within-country (country-mean centered) and between-country (grand-mean centered country averages) estimate for each predictor variable in each model. In addition, all control variables were included as fixed effects. Canada (French speaking and English speaking), China (China and Hong Kong), United Kingdom (England, Northern Ireland, Scotland, and Wales), and United States (North and South) samples were collected from different locations and were treated as separate countries for the sake of analyses.

## Supplementary Material

pgae221_Supplementary_Data

## Data Availability

Original and secondary data were used in the current article. Data for Study 1 and Study 2 are available on the Open Science Framework: https://osf.io/3mhs8/?view_only=13a8c5a4838942829b86397fdf97a19a. Further use of the multinational data from Study 2 for future publications is restricted, and the Data Use Agreement can be found on the OSF link provided above. Data for Study 1 was downloaded from TwitPlat using Kibana version 7.5.1. All analyses were performed on R studio version 2022.07.0. All R code, including the packages used, can be found on the Open Science Framework via the links provided above.

## Footnotes

1 Liberty/oppression words were not available in the Moral Foundations Dictionary.

2 Models would only converge with random slopes when the random effects structure was simplified. All random slopes models contained place code and year as crossed random effects, whereas county and state were no longer included as grouping variables.

3 For the sake of simplicity, we refer to the 41 locations as “countries” throughout. However, we acknowledge that several of our samples came from different regions within the same country (e.g. Canada, China, United Kingdom, and United States).

4 One country was excluded from analyses due to complete missing data on a critical control variable, subjective social status.

5 As the variables from this study came from a larger multinational survey, the hypotheses were devised after data collection. The variables were not given to participants in the hypothesized order (the dependent variable, moral judgments, was measured before the mediator, perceptions of anomie), and we note this as a possible limitation.

6 A third item assessing purity (i.e. “You see a man having sex with a frozen chicken before cooking it for dinner”) was initially included in the survey but was removed in several locations and was therefore dropped from analyses.

7 Our survey contained a third measure of economic inequality—explicit perceptions of how large the wealth gap is between the rich and the poor (5). We have included details on this measure as well as the relationship between this measure and moral judgments in the Supplementary Materials. Broadly speaking, this measure predicted moral judgments in a similar way to the country-level and perceived Gini Index.

## Preprint

This manuscript was posted as a preprint: https://doi.org/10.31234/osf.io/5dnt8.
