# Supplementary material for: High economic inequality is linked to greater moralization
Source: PNAS Nexus. 2024 Jun 5;3(7):pgae221. doi: 10.1093/pnasnexus/pgae221 (PMC11229818; doi:10.1093/pnasnexus/pgae221)
Supplement: pgae221_Supplementary_Data [file pgae221_supplementary_data.docx]

**Supplementary Materials**

Table of Contents

[Supplementary Materials 1: Full Model Results for Study 1 2](#_Toc167972270)

[Supplementary Materials 2: Full Model Results for Study 2 18](#_Toc167972271)

[Supplementary Materials 3: Figures for Average Judgment Scores per Country 30](#_Toc167972272)

[Supplementary Materials 4: Correlations for Variables in Study 2 41](#_Toc167972273)

[Supplementary Materials 5: Mediation Results for Study 2 42](#_Toc167972274)

[Supplementary Materials 6: Amended Dictionary for Study 1 43](#_Toc167972275)

[Supplementary Materials 7: Information About Multinational Sample in Study 2 45](#_Toc167972276)

[Supplementary Materials 8: Measures for Study 2 46](#_Toc167972277)

[Supplementary Materials 9: Wealth Gap Results for Study 2 49](#_Toc167972278)

[References 56](#_Toc167972279)

# Supplementary Materials 1: Full Model Results for Study 1

Below we do not report ICCs and Marginal R^2^ / Conditional R^2^ due to model processing power required to calculate these. However, the script, code and data has been made available on OSF (<https://osf.io/3mhs8/?view_only=13a8c5a4838942829b86397fdf97a19a>) if researchers wish to calculate these.

Table S1

*Results for AIC Comparison for Random Effects Structure with Intercept Only Models*

| Random Effects Structure | AIC |
| --- | --- |
| (1\|Year) + (1\|StateCode/CountyCode/PlaceCode) | 479545.2 |
| (1\|Year) + (1\|CountyCode/PlaceCode) | 479589.9 |
| (1\|Year) + (1\|StateCode/PlaceCode) | 479624.2 |
| (1\|Year) + (1\|PlaceCode) | 482058.8 |
| (1\|Year) + (1\|CountyCode) | 556437.1 |
| (1\|Year) + (1\|StateCode) | 568455.3 |

An ANOVA revealed that the AIC for the first random effects structure (*[1|Year] + [1|StateCode/CountyCode/PlaceCode]*) was significantly lower relative to the second random effects structure (*[1|Year] + [1|CountyCode/PlaceCode]*), χ^2^(1) = 46.70, *p* < .001.

**Main Findings**

Table S2

*Negative Binomial Generalized Linear Mixed Model Examining the Effect of Gini Index on Use of Moral Words (Total)*

|  | **Total Moral Word Use** | | |
| --- | --- | --- | --- |
| *Predictors* | *Estimates* | *SE* | *p* |
| (Intercept) | 1.27 |  | <.001 |
| Gini Index | 0.63 |  | <.001 |
| **Random Effects** |  | | |
| Place/County/State | 0.71 | | |
| County/State | 0.04 | | |
| State | 0.02 | | |
| Year | 0.02 | | |
| N (Place) | 5403 | | |
| N (County) | 1569 | | |
| N (State) | 50 | | |
| N (Year) | 9 | | |
| Observations | 46778 | | |

Table SS3

*Negative Binomial Generalized Linear Mixed Model Examining the Effect of Gini Index on Use of Virtue Words*

|  | **Virtue Word Use** | | |
| --- | --- | --- | --- |
| *Predictors* | *Estimates* | *SE* | *p* |
| (Intercept) | 0.65 | 0.07 | <.001 |
| Gini Index | 0.56 | 0.08 | <.001 |
| **Random Effects** |  | | |
| Place/County/State | 0.71 | | |
| County/State | 0.04 | | |
| State | 0.02 | | |
| Year | 0.02 | | |
| N (Place) | 5403 | | |
| N (County) | 1569 | | |
| N (State) | 50 | | |
| N (Year) | 9 | | |
| Observations | 46778 | | |

Table S4

*Negative Binomial Generalized Linear Mixed Model Examining the Effect of Gini Index on Use of Vice Words*

|  | **Vice Word Use** | | |
| --- | --- | --- | --- |
| *Predictors* | *Estimates* | *SE* | *p* |
| (Intercept) | 0.16 | 0.07 | .019 |
| Gini Index | 0.91 | 0.09 | <.001 |
| **Random Effects** |  | | |
| Place/County/State | 0.68 | | |
| County/State | 0.04 | | |
| State | 0.02 | | |
| Year | 0.02 | | |
| N (Place) | 5403 | | |
| N (County) | 1569 | | |
| N (State) | 50 | | |
| N (Year) | 9 | | |
| Observations | 46778 | | |

Table S5

*Negative Binomial Generalized Linear Mixed Model Examining the Effect of Gini Index on Use of Individualizing Words*

|  | **Individualizing Word Use** | | |
| --- | --- | --- | --- |
| *Predictors* | *Estimates* | *SE* | *p* |
| (Intercept) | 0.59 | 0.06 | <.001 |
| Gini Index | 0.69 | 0.08 | <.001 |
| **Random Effects** |  | | |
| Place/County/State | 0.70 | | |
| County/State | 0.04 | | |
| State | 0.02 | | |
| Year | 0.02 | | |
| N (Place) | 5403 | | |
| N (County) | 1569 | | |
| N (State) | 50 | | |
| N (Year) | 9 | | |
| Observations | 46778 | | |

Table S6

*Negative Binomial Generalized Linear Mixed Model Examining the Effect of Gini Index on Use of Binding Words*

|  | **Binding Word Use** | | |
| --- | --- | --- | --- |
| *Predictors* | *Estimates* | *SE* | *p* |
| (Intercept) | 0.24 | 0.08 | .002 |
| Gini Index | 0.74 | 0.08 | <.001 |
| **Random Effects** |  | | |
| Place/County/State | 0.70 | | |
| County/State | 0.04 | | |
| State | 0.02 | | |
| Year | 0.03 | | |
| N (Place) | 5403 | | |
| N (County) | 1569 | | |
| N (State) | 50 | | |
| N (Year) | 9 | | |
| Observations | 46778 | | |

Table S7

*Negative Binomial Generalized Linear Mixed Model Examining the Effect of Gini Index on Use of Harm Words*

|  | **Harm Word Use** | | |
| --- | --- | --- | --- |
| *Predictors* | *Estimates* | *SE* | *p* |
| (Intercept) | 0.41 | 0.06 | <.001 |
| Gini Index | 0.70 | 0.08 | <.001 |
| **Random Effects** |  | | |
| Place/County/State | 0.69 | | |
| County/State | 0.04 | | |
| State | 0.02 | | |
| Year | 0.02 | | |
| N (Place) | 5403 | | |
| N (County) | 1569 | | |
| N (State) | 50 | | |
| N (Year) | 9 | | |
| Observations | 46778 | | |

Table S8

*Negative Binomial Generalized Linear Mixed Model Examining the Effect of Gini Index on Use of Fairness Words*

|  | **Fairness Word Use** | | |
| --- | --- | --- | --- |
| *Predictors* | *Estimates* | *SE* | *p* |
| (Intercept) | -1.44 | 0.08 | <.001 |
| Gini Index | 1.29 | 0.11 | <.001 |
| **Random Effects** |  | | |
| Place/County/State | 0.66 | | |
| County/State | 0.04 | | |
| State | 0.02 | | |
| Year | 0.03 | | |
| N (Place) | 5403 | | |
| N (County) | 1569 | | |
| N (State) | 50 | | |
| N (Year) | 9 | | |
| Observations | 46778 | | |

Table S9

*Negative Binomial Generalized Linear Mixed Model Examining the Effect of Gini Index on Use of Loyalty Words*

|  | **Loyalty Word Use** | | |
| --- | --- | --- | --- |
| *Predictors* | *Estimates* | *SE* | *p* |
| (Intercept) | -0.64 | 0.09 | <.001 |
| Gini Index | 1.00 | 0.10 | <.001 |
| **Random Effects** |  | | |
| Place/County/State | 0.69 | | |
| County/State | 0.04 | | |
| State | 0.02 | | |
| Year | 0.05 | | |
| N (Place) | 5403 | | |
| N (County) | 1569 | | |
| N (State) | 50 | | |
| N (Year) | 9 | | |
| Observations | 46778 | | |

Table S10

*Negative Binomial Generalized Linear Mixed Model Examining the Effect of Gini Index on Use of Authority Words*

|  | **Authority Word Use** | | |
| --- | --- | --- | --- |
| *Predictors* | *Estimates* | *SE* | *p* |
| (Intercept) | -1.26 | 0.10 | <.001 |
| Gini Index | 1.20 | 0.11 | <.001 |
| **Random Effects** |  | | |
| Place/County/State | 0.65 | | |
| County/State | 0.03 | | |
| State | 0.02 | | |
| Year | 0.07 | | |
| N (Place) | 5403 | | |
| N (County) | 1569 | | |
| N (State) | 50 | | |
| N (Year) | 9 | | |
| Observations | 46778 | | |

Table S11

*Negative Binomial Generalized Linear Mixed Model Examining the Effect of Gini Index on Use of Purity Words*

|  | **Purity Word Use** | | |
| --- | --- | --- | --- |
| *Predictors* | *Estimates* | *SE* | *p* |
| (Intercept) | -1.18 | 0.07 | <.001 |
| Gini Index | 0.96 | 0.10 | <.001 |
| **Random Effects** |  | | |
| Place/County/State | 0.66 | | |
| County/State | 0.04 | | |
| State | 0.02 | | |
| Year | 0.03 | | |
| N (Place) | 5403 | | |
| N (County) | 1569 | | |
| N (State) | 50 | | |
| N (Year) | 9 | | |
| Observations | 46778 | | |

**Models with Random Slopes**

Table S12

*Negative Binomial Generalized Linear Mixed Model Examining the Effect of Gini Index on Use of Moral Words (Total) with Random Slopes*

|  | **Total Moral Word Use** | | |
| --- | --- | --- | --- |
| *Predictors* | *Estimates* | *SE* | *p* |
| (Intercept) | 1.03 | 0.08 | <.001 |
| Gini Index | 1.08 | 0.13 | <.001 |
| **Random Effects** |  | | |
| Place (intercept) | 6.62 | | |
| Place (Gini) | 36.12 | | |
| Year (intercept) | 0.03 | | |
| Year (Gini) | 0.02 | | |
| N (Place) | 5433 | | |
| N (Year) | 9 | | |
| Observations | 46954 | | |

Table S13

*Negative Binomial Generalized Linear Mixed Model Examining the Effect of Gini Index on Use of Virtue Words with Random Slopes*

|  | **Virtue Word Use** | | |
| --- | --- | --- | --- |
| *Predictors* | *Estimates* | *SE* | *p* |
| (Intercept) | 0.42 | 0.09 | <.001 |
| Gini Index | 0.98 | 0.13 | <.001 |
| **Random Effects** |  | | |
| Place (intercept) | 5.96 | | |
| Place (Gini) | 31.73 | | |
| Year (intercept) | 0.04 | | |
| Year (Gini) | 0.03 | | |
| N (Place) | 5433 | | |
| N (Year) | 9 | | |
| Observations | 46954 | | |

Table S14

*Negative Binomial Generalized Linear Mixed Model Examining the Effect of Gini Index on Use of Vice Words with Random Slopes*

|  | **Vice Word Use** | | |
| --- | --- | --- | --- |
| *Predictors* | *Estimates* | *SE* | *p* |
| (Intercept) | -0.09 | 0.08 | .280 |
| Gini Index | 1.41 | 0.15 | <.001 |
| **Random Effects** |  | | |
| Place (intercept) | 7.95 | | |
| Place (Gini) | 44.37 | | |
| Year (intercept) | 0.03 | | |
| Year (Gini) | 0.02 | | |
| N (Place) | 5433 | | |
| N (Year) | 9 | | |
| Observations | 46954 | | |

Table S15

*Negative Binomial Generalized Linear Mixed Model Examining the Effect of Gini Index on Use of Individualizing Words with Random Slopes*

|  | **Individualizing Word Use** | | |
| --- | --- | --- | --- |
| *Predictors* | *Estimates* | *SE* | *p* |
| (Intercept) | 0.36 | 0.07 | <.001 |
| Gini Index | 1.12 | 0.13 | <.001 |
| **Random Effects** |  | | |
| Place (intercept) | 6.60 | | |
| Place (Gini) | 36.43 | | |
| Year (intercept) | 0.02 | | |
| Year (Gini) | 0.02 | | |
| N (Place) | 5433 | | |
| N (Year) | 9 | | |
| Observations | 46954 | | |

Table S16

*Negative Binomial Generalized Linear Mixed Model Examining the Effect of Gini Index on Use of Binding Words with Random Slopes*

|  | **Binding Word Use** | | |
| --- | --- | --- | --- |
| *Predictors* | *Estimates* | *SE* | *p* |
| (Intercept) | -0.01 | 0.10 | .904 |
| Gini Index | 1.22 | 0.15 | <.001 |
| **Random Effects** |  | | |
| Place (intercept) | 6.86 | | |
| Place (Gini) | 37.32 | | |
| Year (intercept) | 0.07 | | |
| Year (Gini) | 0.04 | | |
| N (Place) | 5433 | | |
| N (Year) | 9 | | |
| Observations | 46954 | | |

Table S17

*Negative Binomial Generalized Linear Mixed Model Examining the Effect of Gini Index on Use of Harm Words with Random Slopes*

|  | **Harm Word Use** | | |
| --- | --- | --- | --- |
| *Predictors* | *Estimates* | *SE* | *p* |
| (Intercept) | 0.19 | 0.07 | .009 |
| Gini Index | 1.11 | 0.13 | <.001 |
| **Random Effects** |  | | |
| Place (intercept) | 6.33 | | |
| Place (Gini) | 35.07 | | |
| Year (intercept) | 0.02 | | |
| Year (Gini) | 0.01 | | |
| N (Place) | 5433 | | |
| N (Year) | 9 | | |
| Observations | 46954 | | |

Table S18

*Negative Binomial Generalized Linear Mixed Model Examining the Effect of Gini Index on Use of Fairness Words with Random Slopes*

|  | **Fairness Word Use** | | |
| --- | --- | --- | --- |
| *Predictors* | *Estimates* | *SE* | *p* |
| (Intercept) | -1.71 | 0.10 | <.001 |
| Gini Index | 1.84 | 0.18 | <.001 |
| **Random Effects** |  | | |
| Place (intercept) | 10.05 | | |
| Place (Gini) | 53.27 | | |
| Year (intercept) | 0.04 | | |
| Year (Gini) | 0.05 | | |
| N (Place) | 5433 | | |
| N (Year) | 9 | | |
| Observations | 46954 | | |

*Note:* Model failed to converge

Table S19

*Negative Binomial Generalized Linear Mixed Model Examining the Effect of Gini Index on Use of Loyalty Words with Random Slopes*

|  | **Loyalty Word Use** | | |
| --- | --- | --- | --- |
| *Predictors* | *Estimates* | *SE* | *p* |
| (Intercept) | -0.87 | 0.13 | <.001 |
| Gini Index | 1.44 | 0.18 | <.001 |
| **Random Effects** |  | | |
| Place (intercept) | 7.25 | | |
| Place (Gini) | 39.63 | | |
| Year (intercept) | 0.12 | | |
| Year (Gini) | 0.09 | | |
| N (Place) | 5433 | | |
| N (Year) | 9 | | |
| Observations | 46954 | | |

Table S20

*Negative Binomial Generalized Linear Mixed Model Examining the Effect of Gini Index on Use of Authority Words with Random Slopes*

|  | **Authority Word Use** | | |
| --- | --- | --- | --- |
| *Predictors* | *Estimates* | *SE* | *p* |
| (Intercept) | -1.49 | 0.15 | <.001 |
| Gini Index | 1.70 | 0.20 | <.001 |
| **Random Effects** |  | | |
| Place (intercept) | 7.34 | | |
| Place (Gini) | 39.02 | | |
| Year (intercept) | 0.17 | | |
| Year (Gini) | 0.14 | | |
| N (Place) | 5433 | | |
| N (Year) | 9 | | |
| Observations | 46954 | | |

Table S21

*Negative Binomial Generalized Linear Mixed Model Examining the Effect of Gini Index on Use of Purity Words with Random Slopes*

|  | **Purity Word Use** | | |
| --- | --- | --- | --- |
| *Predictors* | *Estimates* | *SE* | *p* |
| (Intercept) | -1.38 | 0.10 | <.001 |
| Gini Index | 1.34 | 0.16 | <.001 |
| **Random Effects** |  | | |
| Place (intercept) | 6.05 | | |
| Place (Gini) | 32.89 | | |
| Year (intercept) | 0.06 | | |
| Year (Gini) | 0.03 | | |
| N (Place) | 5433 | | |
| N (Year) | 9 | | |
| Observations | 46954 | | |

*Note:* Model failed to converge

**Models with Controls**

Table S22

*Negative Binomial Generalized Linear Mixed Model Examining the Effect of Gini Index on Use of Moral Words (Total) Controlling for Variables*

|  | **Total Moral Word Use** | | |
| --- | --- | --- | --- |
| *Predictors* | *Estimates* | *SE* | *p* |
| (Intercept) | 0.98 | 0.14 | <.001 |
| Gini Index | 0.61 | 0.07 | <.001 |
| Religiosity | 0.83 | 0.23 | <.001 |
| Voting behavior | -0.13 | 0.02 | <.001 |
| GDP | 0.25 | 0.02 | <.001 |
| **Random Effects** |  | | |
| Place/County/State | 0.70 | | |
| County/State | 0.06 | | |
| State | 0.01 | | |
| Year | 0.02 | | |
| N (Place) | 5403 | | |
| N (County) | 1569 | | |
| N (State) | 50 | | |
| N (Year) | 9 | | |
| Observations | 46778 | | |

Table S23

*Negative Binomial Generalized Linear Mixed Model Examining the Effect of Gini Index on Use of Virtue Words Controlling for Variables*

|  | **Virtue Word Use** | | |
| --- | --- | --- | --- |
| *Predictors* | *Estimates* | *SE* | *p* |
| (Intercept) | 0.37 | 0.14 | .009 |
| Gini Index | 0.54 | 0.07 | <.001 |
| Religiosity | 0.77 | 0.24 | .001 |
| Voting behavior | -0.11 | 0.02 | <.001 |
| GDP | 0.23 | 0.02 | <.001 |
| **Random Effects** |  | | |
| Place/County/State | 0.71 | | |
| County/State | 0.06 | | |
| State | 0.01 | | |
| Year | 0.02 | | |
| N (Place) | 5403 | | |
| N (County) | 1569 | | |
| N (State) | 50 | | |
| N (Year) | 9 | | |
| Observations | 46778 | | |

Table S24

*Negative Binomial Generalized Linear Mixed Model Examining the Effect of Gini Index on Use of Vice Words Controlling for Variables*

|  | **Vice Word Use** | | |
| --- | --- | --- | --- |
| *Predictors* | *Estimates* | *SE* | *p* |
| (Intercept) | -0.07 | 0.14 | .602 |
| Gini Index | 0.89 | 0.09 | <.001 |
| Religiosity | 0.74 | 0.22 | <.001 |
| Voting behavior | -0.16 | 0.03 | <.001 |
| GDP | 0.23 | 0.02 | <.001 |
| **Random Effects** |  | | |
| Place/County/State | 0.68 | | |
| County/State | 0.06 | | |
| State | 0.01 | | |
| Year | 0.02 | | |
| N (Place) | 5403 | | |
| N (County) | 1569 | | |
| N (State) | 50 | | |
| N (Year) | 9 | | |
| Observations | 46778 | | |

Table S25

*Negative Binomial Generalized Linear Mixed Model Examining the Effect of Gini Index on Use of Individualizing Words Controlling for Variables*

|  | **Individualizing Word Use** | | |
| --- | --- | --- | --- |
| *Predictors* | *Estimates* | *SE* | *p* |
| (Intercept) | 0.36 | 0.14 | .009 |
| Gini Index | 0.67 | 0.08 | <.001 |
| Religiosity | 0.74 | 0.23 | .001 |
| Voting behavior | -0.13 | 0.02 | <.001 |
| GDP | 0.26 | 0.02 | <.001 |
| **Random Effects** |  | | |
| Place/County/State | 0.69 | | |
| County/State | 0.06 | | |
| State | 0.01 | | |
| Year | 0.02 | | |
| N (Place) | 5403 | | |
| N (County) | 1569 | | |
| N (State) | 50 | | |
| N (Year) | 9 | | |
| Observations | 46778 | | |

Table S26

*Negative Binomial Generalized Linear Mixed Model Examining the Effect of Gini Index on Use of Binding Words Controlling for Variables*

|  | **Binding Word Use** | | |
| --- | --- | --- | --- |
| *Predictors* | *Estimates* | *SE* | *p* |
| (Intercept) | -0.05 | 0.14 | .738 |
| Gini Index | 0.73 | 0.08 | <.001 |
| Religiosity | 0.77 | 0.23 | <.001 |
| Voting behavior | -0.12 | 0.02 | <.001 |
| GDP | 0.20 | 0.02 | <.001 |
| **Random Effects** |  | | |
| Place/County/State | 0.69 | | |
| County/State | 0.06 | | |
| State | 0.01 | | |
| Year | 0.03 | | |
| N (Place) | 5403 | | |
| N (County) | 1569 | | |
| N (State) | 50 | | |
| N (Year) | 9 | | |
| Observations | 46778 | | |

Table S27

*Negative Binomial Generalized Linear Mixed Model Examining the Effect of Gini Index on Use of Harm Words Controlling for Variables*

|  | **Harm Word Use** | | |
| --- | --- | --- | --- |
| *Predictors* | *Estimates* | *SE* | *p* |
| (Intercept) | 0.13 | 0.13 | .344 |
| Gini Index | 0.68 | 0.08 | <.001 |
| Religiosity | 0.80 | 0.22 | <.001 |
| Voting behavior | -0.12 | 0.02 | <.001 |
| GDP | 0.24 | 0.02 | <.001 |
| **Random Effects** |  | | |
| Place/County/State | 0.68 | | |
| County/State | 0.06 | | |
| State | 0.01 | | |
| Year | 0.02 | | |
| N (Place) | 5403 | | |
| N (County) | 1569 | | |
| N (State) | 50 | | |
| N (Year) | 9 | | |
| Observations | 46778 | | |

Table S28

*Negative Binomial Generalized Linear Mixed Model Examining the Effect of Gini Index on Use of Fairness Words Controlling for Variables*

|  | **Fairness Word Use** | | |
| --- | --- | --- | --- |
| *Predictors* | *Estimates* | *SE* | *p* |
| (Intercept) | -1.39 | 0.15 | <.001 |
| Gini Index | 1.28 | 0.11 | <.001 |
| Religiosity | 0.25 | 0.25 | .307 |
| Voting behavior | -0.19 | 0.03 | <.001 |
| GDP | 0.23 | 0.02 | <.001 |
| **Random Effects** |  | | |
| Place/County/State | 0.66 | | |
| County/State | 0.06 | | |
| State | 0.02 | | |
| Year | 0.03 | | |
| N (Place) | 5403 | | |
| N (County) | 1569 | | |
| N (State) | 50 | | |
| N (Year) | 9 | | |
| Observations | 46778 | | |

Table S29

*Negative Binomial Generalized Linear Mixed Model Examining the Effect of Gini Index on Use of Loyalty Words Controlling for Variables*

|  | **Loyalty Word Use** | | |
| --- | --- | --- | --- |
| *Predictors* | *Estimates* | *SE* | *p* |
| (Intercept) | -0.76 | 0.15 | <.001 |
| Gini Index | 0.99 | 0.10 | <.001 |
| Religiosity | 0.38 | 0.23 | .108 |
| Voting behavior | -0.08 | 0.03 | .003 |
| GDP | 0.14 | 0.02 | <.001 |
| **Random Effects** |  | | |
| Place/County/State | 0.68 | | |
| County/State | 0.05 | | |
| State | 0.01 | | |
| Year | 0.05 | | |
| N (Place) | 5403 | | |
| N (County) | 1569 | | |
| N (State) | 50 | | |
| N (Year) | 9 | | |
| Observations | 46778 | | |

Table S30

*Negative Binomial Generalized Linear Mixed Model Examining the Effect of Gini Index on Use of Authority Words Controlling for Variables*

|  | **Authority Word Use** | | |
| --- | --- | --- | --- |
| *Predictors* | *Estimates* | *SE* | *p* |
| (Intercept) | -1.66 | 0.15 | <.001 |
| Gini Index | 1.20 | 0.11 | <.001 |
| Religiosity | 0.92 | 0.22 | <.001 |
| Voting behavior | -0.09 | 0.03 | .002 |
| GDP | 0.15 | 0.02 | <.001 |
| **Random Effects** |  | | |
| Place/County/State | 0.64 | | |
| County/State | 0.04 | | |
| State | 0.01 | | |
| Year | 0.07 | | |
| N (Place) | 5403 | | |
| N (County) | 1569 | | |
| N (State) | 50 | | |
| N (Year) | 9 | | |
| Observations | 46778 | | |

Table S31

*Negative Binomial Generalized Linear Mixed Model Examining the Effect of Gini Index on Use of Purity Words Controlling for Variables*

|  | **Purity Word Use** | | |
| --- | --- | --- | --- |
| *Predictors* | *Estimates* | *SE* | *p* |
| (Intercept) | -1.50 | 0.14 | <.001 |
| Gini Index | 0.94 | 0.10 | <.001 |
| Religiosity | 0.97 | 0.23 | <.001 |
| Voting behavior | -0.21 | 0.03 | <.001 |
| GDP | 0.23 | 0.02 | <.001 |
| **Random Effects** |  | | |
| Place/County/State | 0.65 | | |
| County/State | 0.06 | | |
| State | 0.01 | | |
| Year | 0.03 | | |
| N (Place) | 5403 | | |
| N (County) | 1569 | | |
| N (State) | 50 | | |
| N (Year) | 9 | | |
| Observations | 46778 | | |

**Cross-Lagged Models**

Table S32

*Cross Lagged Negative Binomial Generalized Linear Mixed Model Examining the Effect of Gini Index at Time 1 on Use of Moral Words (Total) at Time 2*

|  | **Total Moral Word Use (Time 2)** | | |
| --- | --- | --- | --- |
| *Predictors* | *Estimates* | *SE* | *p* |
| (Intercept) | 1.05 | 0.07 | <.001 |
| Gini Index Time 1 | 0.61 | 0.11 | <.001 |
| Gini Index Time 2 | 0.32 | 0.10 | .002 |
| Total Moral Words (Time 1) | 1.33 | 0.07 | <.001 |
| **Random Effects** |  | | |
| Place/County/State | 0.70 | | |
| County/State | 0.04 | | |
| State | 0.02 | | |
| Year | 0.02 | | |
| N (Place) | 5372 | | |
| N (County) | 1569 | | |
| N (State) | 50 | | |
| N (Year) | 9 | | |
| Observations | 41373 | | |

# Supplementary Materials 2: Full Model Results for Study 2

**Results for Perceived Gini Index**

Table S33

*Linear Mixed Model Examining the Effect of Country Gini Index on Total Moral Judgment Score*

|  | **Total Moral Judgments** | | |
| --- | --- | --- | --- |
| *Predictors* | *Estimates* | *CI* | *p* |
| (Intercept) | -0.21 | -0.33, -0.09 | .001 |
| Gini Index (World Bank) | 0.18 | 0.04, 0.32 | .011 |
| GDP | -0.04 | -0.16, 0.09 | .543 |
| Age | 0.03 | 0.01, 0.06 | .017 |
| Gender [female] | 0.37 | 0.31, 0.42 | <.001 |
| Economic conservativism | -0.04 | -0.07, -0.01 | .012 |
| Social conservativism | 0.04 | 0.01, 0.07 | .005 |
| Subjective social status | -0.03 | -0.06, -0.01 | .010 |
| Importance of religion | 0.10 | 0.07, 0.13 | <.001 |
| **Random Effects** | | | |
| Residual | 0.82 | | |
| Country (intercept) | 0.10 | | |
| ICC | .11 | | |
| N (country) | 38 | | |
| Observations | 5581 | | |
| Marginal R2 / Conditional R2 | .075 / .179 | | |

*Note:* Gender was coded as male (1) and female (2).

Table S34

*Linear Mixed Model Examining the Effect of Country Gini Index on Individualising Moral Judgment Score*

|  | **Individualising** | | |
| --- | --- | --- | --- |
| *Predictors* | *Estimates* | *CI* | *p* |
| (Intercept) | -0.14 | -0.27, -0.01 | .034 |
| Gini Index (World Bank) | 0.17 | 0.02, 0.32 | .026 |
| GDP | 0.02 | -0.12, 0.16 | .781 |
| Age | 0.02 | -0.01, 0.05 | .112 |
| Gender [female] | 0.33 | 0.27, 0.38 | <.001 |
| Economic conservativism | -0.05 | -0.08, -0.02 | <.001 |
| Social conservativism | -0.03 | -0.06, -0.00 | .027 |
| Subjective social status | -0.01 | -0.04, 0.01 | .378 |
| Importance of religion | 0.01 | -0.02, 0.04 | .621 |
| **Random Effects** | | | |
| Residual | 0.78 | | |
| Country (intercept) | 0.13 | | |
| ICC | .14 | | |
| N (country) | 38 | | |
| Observations | 5599 | | |
| Marginal R2 / Conditional R2 | .051 / .188 | | |

*Note:* Gender was coded as male (1) and female (2).

Table S35

*Linear Mixed Model Examining the Effect of Country Gini Index on Binding Moral Judgment Score*

|  | **Binding** | | |
| --- | --- | --- | --- |
| *Predictors* | *Estimates* | *CI* | *p* |
| (Intercept) | -0.15 | -0.27, -0.03 | .016 |
| Gini Index (World Bank) | 0.16 | 0.02, 0.30 | .024 |
| GDP | -0.07 | -0.20, 0.05 | .247 |
| Age | 0.04 | 0.01, 0.06 | .008 |
| Gender [female] | 0.18 | 0.13, 0.24 | <.001 |
| Economic conservativism | 0.01 | -0.02, 0.03 | .714 |
| Social conservativism | 0.15 | 0.12, 0.18 | <.001 |
| Subjective social status | -0.05 | -0.07, -0.02 | <.001 |
| Importance of religion | 0.18 | 0.15, 0.21 | **<.001** |
| **Random Effects** |  | | |
| Residual | 0.74 | | |
| Country (intercept) | 0.11 | | |
| ICC | .13 | | |
| N (country) | 38 | | |
| Observations | 5604 | | |
| Marginal R^2^ / Conditional R^2^ | .147 / .258 | | |

*Note:* Gender was coded as male (1) and female (2).

Table S36

*Linear Mixed Model Examining the Effect of Country Gini Index on Harm Moral Judgment Score*

|  | **Harm** | | |
| --- | --- | --- | --- |
| *Predictors* | *Estimates* | *CI* | *p* |
| (Intercept) | -0.21 | -0.34, -0.08 | .003 |
| Gini Index (World Bank) | 0.06 | -0.09, 0.22 | .404 |
| GDP | 0.00 | -0.14, 0.14 | .993 |
| Age | -0.02 | -0.04, 0.01 | .238 |
| Gender [female] | 0.41 | 0.36, 0.46 | <.001 |
| Economic conservativism | -0.06 | -0.09, -0.03 | <.001 |
| Social conservativism | -0.04 | -0.07, -0.01 | .007 |
| Subjective social status | -0.00 | -0.03, 0.02 | .858 |
| Importance of religion | 0.02 | -0.01, 0.04 | .290 |
| **Random Effects** | | | |
| Residual | 0.78 | | |
| Country (intercept) | 0.14 | | |
| ICC | .15 | | |
| N (country) | 38 | | |
| Observations | 5613 | | |
| Marginal R2 / Conditional R2 | .049 / .192 | | |

*Note:* Gender was coded as male (1) and female (2).

Table S37

*Linear Mixed Model Examining the Effect of Country Gini Index on Fairness Moral Judgment Score*

|  | **Fairness** | | |
| --- | --- | --- | --- |
| *Predictors* | *Estimates* | *CI* | *p* |
| (Intercept) | -0.08 | -0.18, 0.02 | .127 |
| Gini Index (World Bank) | 0.19 | 0.07, 0.30 | .002 |
| GDP | -0.07 | -0.17, 0.04 | .214 |
| Age | 0.05 | 0.02, 0.07 | .001 |
| Gender [female] | 0.18 | 0.12, 0.23 | <.001 |
| Economic conservativism | -0.04 | -0.07, -0.01 | .010 |
| Social conservativism | 0.04 | 0.01, 0.07 | .009 |
| Subjective social status | -0.01 | -0.03, 0.02 | .610 |
| Importance of religion | 0.08 | 0.05, 0.11 | <.001 |
| **Random Effects** | | | |
| Residual | 0.84 | | |
| Country (intercept) | 0.07 | | |
| ICC | .08 | | |
| N (country) | 38 | | |
| Observations | 5618 | | |
| Marginal R2 / Conditional R2 | .072 / .147 | | |

*Note:* Gender was coded as male (1) and female (2).

Table S38

*Linear Mixed Model Examining the Effect of Country Gini Index on Liberty Moral Judgment Score*

|  | **Liberty** | | |
| --- | --- | --- | --- |
| *Predictors* | *Estimates* | *CI* | *p* |
| (Intercept) | -0.07 | -0.21, 0.08 | .357 |
| Gini Index (World Bank) | 0.15 | -0.01, 0.32 | .063 |
| GDP | 0.11 | -0.05, 0.26 | .165 |
| Age | 0.02 | -0.01, 0.05 | .183 |
| Gender [female] | 0.22 | 0.16, 0.27 | <.001 |
| Economic conservativism | -0.03 | -0.06, 0.00 | .053 |
| Social conservativism | -0.07 | -0.10, -0.04 | <.001 |
| Subjective social status | -0.01 | -0.04, 0.01 | .231 |
| Importance of religion | -0.07 | -0.10, -0.04 | <.001 |
| **Random Effects** | | | |
| Residual | 0.78 | | |
| Country (intercept) | 0.15 | | |
| ICC | .17 | | |
| N (country) | 38 | | |
| Observations | 5613 | | |
| Marginal R2 / Conditional R2 | .051 / .208 | | |

*Note:* Gender was coded as male (1) and female (2).

Table S39

*Linear Mixed Model Examining the Effect of Country Gini Index on Authority Moral Judgment Score*

|  | **Authority** | | |
| --- | --- | --- | --- |
| *Predictors* | *Estimates* | *CI* | *p* |
| (Intercept) | -0.14 | -0.25, -0.04 | .010 |
| Gini Index (World Bank) | 0.18 | 0.06, 0.30 | .005 |
| GDP | -0.13 | -0.25, -0.02 | .020 |
| Age | 0.05 | 0.02, 0.07 | .001 |
| Gender [female] | 0.20 | 0.15, 0.26 | <.001 |
| Economic conservativism | 0.00 | -0.03, 0.03 | .998 |
| Social conservativism | 0.13 | 0.10, 0.16 | <.001 |
| Subjective social status | -0.08 | -0.10, -0.05 | <.001 |
| Importance of religion | 0.16 | 0.13, 0.18 | <.001 |
| **Random Effects** | | | |
| Residual | 0.73 | | |
| Country (intercept) | 0.08 | | |
| ICC | .10 | | |
| N (country) | 38 | | |
| Observations | 5617 | | |
| Marginal R2 / Conditional R2 | .165 / .250 | | |

*Note:* Gender was coded as male (1) and female (2).

Table S40

*Linear Mixed Model Examining the Effect of Country Gini Index on Loyalty Moral Judgment Score*

|  | **Loyalty** | | |
| --- | --- | --- | --- |
| *Predictors* | *Estimates* | *CI* | *p* |
| (Intercept) | -0.08 | -0.18, 0.03 | .139 |
| Gini Index (World Bank) | 0.11 | -0.01, 0.22 | .063 |
| GDP | -0.11 | -0.22, -0.01 | .034 |
| Age | 0.09 | 0.06, 0.11 | <.001 |
| Gender [female] | 0.07 | 0.02, 0.13 | .012 |
| Economic conservativism | 0.02 | -0.01, 0.05 | .182 |
| Social conservativism | 0.09 | 0.06, 0.12 | <.001 |
| Subjective social status | -0.03 | -0.05, -0.00 | .040 |
| Importance of religion | 0.09 | 0.06, 0.12 | <.001 |
| **Random Effects** | | | |
| Residual | 0.87 | | |
| Country (intercept) | 0.07 | | |
| ICC | .07 | | |
| N (country) | 38 | | |
| Observations | 5614 | | |
| Marginal R2 / Conditional R2 | .082 / .151 | | |

*Note:* Gender was coded as male (1) and female (2).

Table S41

*Linear Mixed Model Examining the Effect of Country Gini Index on Purity Moral Judgment Score*

|  | **Purity** | | |
| --- | --- | --- | --- |
| *Predictors* | *Estimates* | *CI* | *p* |
| (Intercept) | -0.14 | -0.27, -0.01 | .030 |
| Gini Index (World Bank) | 0.11 | -0.04, 0.25 | .138 |
| GDP | 0.05 | -0.08, 0.18 | .454 |
| Age | -0.04 | -0.06, -0.01 | .010 |
| Gender [female] | 0.16 | 0.11, 0.22 | <.001 |
| Economic conservativism | -0.01 | -0.04, 0.02 | .485 |
| Social conservativism | 0.14 | 0.11, 0.17 | <.001 |
| Subjective social status | -0.02 | -0.04, 0.01 | .164 |
| Importance of religion | 0.18 | 0.15, 0.21 | <.001 |
| **Random Effects** | | | |
| Residual | 0.79 | | |
| Country (intercept) | 0.12 | | |
| ICC | .13 | | |
| N (country) | 38 | | |
| Observations | 5619 | | |
| Marginal R2 / Conditional R2 | .084 / .204 | | |

*Note:* Gender was coded as male (1) and female (2).

**Results for Perceived Gini Index**

Table S42

*Linear Mixed Model Examining the Effect of Perceived Gini Index on Total Moral Judgment Score*

|  | **Total Moral Judgments** | | |
| --- | --- | --- | --- |
| *Predictors* | *Estimates* | *CI* | *p* |
| (Intercept) | -0.19 | -0.31, -0.08 | .001 |
| Perceived Gini (between countries) | 0.14 | -0.00, 0.29 | .051 |
| Perceived Gini (within countries) | 0.04 | 0.02, 0.07 | .001 |
| GDP | -0.03 | -0.17, 0.10 | .626 |
| Age | 0.04 | 0.01, 0.07 | .008 |
| Gender [female] | 0.28 | 0.22, 0.33 | <.001 |
| Economic conservativism | -0.03 | -0.05, 0.00 | .088 |
| Social conservativism | 0.08 | 0.05, 0.11 | <.001 |
| Subjective social status | -0.03 | -0.05, -0.00 | .027 |
| Importance of religion | 0.12 | 0.09, 0.15 | **<.001** |
| **Random Effects** |  | | |
| Residual | 0.80 | | |
| Country (intercept) | 0.11 | | |
| ICC | .12 | | |
| N (country) | 41 | | |
| Observations | 5733 | | |
| Marginal R^2^ / Conditional R^2^ | .081 / .189 | | |

*Note:* Gender was coded as male (1) and female (2).

Table S43

*Linear Mixed Model Examining the Effect of Perceived Gini Index on Individualising Moral Judgment Score*

|  | **Individualising** | | |
| --- | --- | --- | --- |
| *Predictors* | *Estimates* | *CI* | *p* |
| (Intercept) | -0.18 | -0.31, -0.05 | .008 |
| Perceived Gini (between countries) | -0.07 | -0.24, 0.10 | .415 |
| Perceived Gini (within countries) | 0.03 | 0.01, 0.05 | .007 |
| GDP | -0.10 | -0.26, 0.06 | .225 |
| Age | 0.02 | -0.01, 0.05 | .164 |
| Gender [female] | 0.31 | 0.26, 0.36 | <.001 |
| Economic conservativism | -0.06 | -0.08, -0.03 | <.001 |
| Social conservativism | -0.04 | -0.06, -0.01 | .019 |
| Subjective social status | -0.00 | -0.03, 0.02 | .770 |
| Importance of religion | 0.00 | -0.02, 0.03 | .802 |
| **Random Effects** |  | | |
| Residual | 0.77 | | |
| Country (intercept) | 0.15 | | |
| ICC | .16 | | |
| N (country) | 41 | | |
| Observations | 5749 | | |
| Marginal R^2^ / Conditional R^2^ | .041 / .199 | | |

*Note:* Gender was coded as male (1) and female (2).

Table S44

*Linear Mixed Model Examining the Effect of Perceived Gini Index on Binding Moral Judgment Score*

|  | **Binding** | | |
| --- | --- | --- | --- |
| *Predictors* | *Estimates* | *CI* | *p* |
| (Intercept) | -0.15 | -0.25, -0.04 | .006 |
| Perceived Gini (between countries) | 0.26 | 0.13, 0.39 | <.001 |
| Perceived Gini (within countries) | 0.03 | 0.01, 0.06 | .002 |
| GDP | 0.03 | -0.10, 0.15 | .680 |
| Age | 0.03 | 0.01, 0.06 | .014 |
| Gender [female] | 0.17 | 0.12, 0.22 | <.001 |
| Economic conservativism | 0.01 | -0.02, 0.03 | .665 |
| Social conservativism | 0.15 | 0.12, 0.18 | <.001 |
| Subjective social status | -0.04 | -0.06, -0.01 | **.003** |
| Importance of religion | 0.17 | 0.14, 0.19 | **<.001** |
| **Random Effects** |  | | |
| Residual | 0.72 | | |
| Country (intercept) | 0.09 | | |
| ICC | .11 | | |
| N (country) | 41 | | |
| Observations | 5749 | | |
| Marginal R^2^ / Conditional R^2^ | .175 / .261 | | |

*Note:* Gender was coded as male (1) and female (2).

Table S45

*Linear Mixed Model Examining the Effect of Perceived Gini Index on Harm Moral Judgment Score*

|  | **Harm** | | |
| --- | --- | --- | --- |
| *Predictors* | *Estimates* | *CI* | *p* |
| (Intercept) | -0.24 | -0.36, -0.11 | <.001 |
| Perceived Gini (between countries) | -0.11 | -0.27, 0.05 | .164 |
| Perceived Gini (within countries) | 0.02 | -0.00, 0.04 | .125 |
| GDP | -0.11 | -0.26, 0.04 | .160 |
| Age | -0.01 | -0.04, 0.01 | .317 |
| Gender [female] | 0.40 | 0.35, 0.45 | <.001 |
| Economic conservativism | -0.06 | -0.09, -0.03 | <.001 |
| Social conservativism | -0.04 | -0.07, -0.01 | .009 |
| Subjective social status | -0.00 | -0.03, 0.02 | .856 |
| Importance of religion | 0.02 | -0.01, 0.04 | .261 |
| **Random Effects** |  | | |
| Residual | 0.75 | | |
| Country (intercept) | 0.13 | | |
| ICC | .15 | | |
| N (country) | 41 | | |
| Observations | 5761 | | |
| Marginal R^2^ / Conditional R^2^ | .064 / .204 | | |

*Note:* Gender was coded as male (1) and female (2).

Table S46

*Linear Mixed Model Examining the Effect of Perceived Gini Index on Fairness Moral Judgment Score*

|  | **Fairness** | | |
| --- | --- | --- | --- |
| *Predictors* | *Estimates* | *CI* | *p* |
| (Intercept) | -0.09 | -0.20, 0.02 | .113 |
| Perceived Gini (between countries) | 0.02 | -0.12, 0.16 | .775 |
| Perceived Gini (within countries) | 0.02 | -0.01, 0.04 | .188 |
| GDP | -0.12 | -0.25, 0.02 | .087 |
| Age | 0.04 | 0.01, 0.07 | .003 |
| Gender [female] | 0.16 | 0.10, 0.21 | <.001 |
| Economic conservativism | -0.05 | -0.08, -0.02 | .002 |
| Social conservativism | 0.04 | 0.01, 0.07 | .022 |
| Subjective social status | 0.00 | -0.02, 0.03 | .947 |
| Importance of religion | 0.08 | 0.05, 0.11 | **<.001** |
| **Random Effects** |  | | |
| Residual | 0.83 | | |
| Country (intercept) | 0.10 | | |
| ICC | .11 | | |
| N (country) | 41 | | |
| Observations | 5768 | | |
| Marginal R^2^ / Conditional R^2^ | .038 / .142 | | |

*Note:* Gender was coded as male (1) and female (2).

Table S47

*Linear Mixed Model Examining the Effect of Perceived Gini Index on Liberty Moral Judgment Score*

|  | **Liberty** | | |
| --- | --- | --- | --- |
| *Predictors* | *Estimates* | *CI* | *p* |
| (Intercept) | -0.11 | -0.25, 0.02 | .102 |
| Perceived Gini (between countries) | -0.08 | -0.26, 0.10 | .367 |
| Perceived Gini (within countries) | 0.04 | 0.02, 0.06 | .001 |
| GDP | -0.02 | -0.19, 0.15 | .795 |
| Age | 0.01 | -0.01, 0.04 | .318 |
| Gender [female] | 0.21 | 0.15, 0.26 | <.001 |
| Economic conservativism | -0.03 | -0.06, -0.00 | .045 |
| Social conservativism | -0.08 | -0.10, -0.05 | <.001 |
| Subjective social status | -0.01 | -0.03, 0.02 | .601 |
| Importance of religion | -0.07 | -0.10, -0.05 | **<.001** |
| **Random Effects** |  | | |
| Residual | 0.77 | | |
| Country (intercept) | 0.17 | | |
| ICC | .18 | | |
| N (country) | 41 | | |
| Observations | 5762 | | |
| Marginal R^2^ / Conditional R^2^ | .049 / .219 | | |

*Note:* Gender was coded as male (1) and female (2).

Table S48

*Linear Mixed Model Examining the Effect of Perceived Gini Index on Authority Moral Judgment Score*

|  | **Authority** | | |
| --- | --- | --- | --- |
| *Predictors* | *Estimates* | *CI* | *p* |
| (Intercept) | -0.14 | -0.24, -0.04 | .008 |
| Perceived Gini (between countries) | 0.19 | 0.06, 0.32 | .006 |
| Perceived Gini (within countries) | 0.02 | -0.00, 0.04 | .090 |
| GDP | -0.08 | -0.21, 0.04 | .190 |
| Age | 0.04 | 0.01, 0.07 | .002 |
| Gender [female] | 0.18 | 0.13, 0.23 | <.001 |
| Economic conservativism | -0.00 | -0.03, 0.03 | .918 |
| Social conservativism | 0.12 | 0.09, 0.15 | <.001 |
| Subjective social status | -0.06 | -0.09, -0.04 | <.001 |
| Importance of religion | 0.15 | 0.12, 0.17 | **<.001** |
| **Random Effects** |  | | |
| Residual | 0.72 | | |
| Country (intercept) | 0.09 | | |
| ICC | .11 | | |
| N (country) | 41 | | |
| Observations | 5764 | | |
| Marginal R^2^ / Conditional R^2^ | .157 / .247 | | |

*Note:* Gender was coded as male (1) and female (2).

Table S49

*Linear Mixed Model Examining the Effect of Perceived Gini Index on Loyalty Moral Judgment Score*

|  | **Loyalty** | | |
| --- | --- | --- | --- |
| *Predictors* | *Estimates* | *CI* | *p* |
| (Intercept) | -0.07 | -0.15, 0.02 | .125 |
| Perceived Gini (between countries) | 0.20 | 0.10, 0.31 | <.001 |
| Perceived Gini (within countries) | 0.05 | 0.02, 0.07 | <.001 |
| GDP | -0.01 | -0.11, 0.09 | .829 |
| Age | 0.09 | 0.06, 0.12 | <.001 |
| Gender [female] | 0.07 | 0.01, 0.13 | .013 |
| Economic conservativism | 0.02 | -0.01, 0.05 | .152 |
| Social conservativism | 0.10 | 0.06, 0.13 | <.001 |
| Subjective social status | -0.02 | -0.04, 0.01 | .149 |
| Importance of religion | 0.08 | 0.05, 0.11 | **<.001** |
| **Random Effects** |  | | |
| Residual | 0.85 | | |
| Country (intercept) | 0.05 | | |
| ICC | .06 | | |
| N (country) | 41 | | |
| Observations | 5761 | | |
| Marginal R^2^ / Conditional R^2^ | .097 / .148 | | |

*Note:* Gender was coded as male (1) and female (2).

Table S50

*Linear Mixed Model Examining the Effect of Perceived Gini Index on Purity Moral Judgment Score*

|  | **Purity** | | |
| --- | --- | --- | --- |
| *Predictors* | *Estimates* | *CI* | *p* |
| (Intercept) | -0.14 | -0.25, -0.03 | .013 |
| Perceived Gini (between countries) | 0.22 | 0.08, 0.36 | .003 |
| Perceived Gini (within countries) | 0.02 | -0.01, 0.04 | .186 |
| GDP | 0.13 | -0.01, 0.26 | .061 |
| Age | -0.04 | -0.07, -0.01 | .008 |
| Gender [female] | 0.15 | 0.10, 0.21 | <.001 |
| Economic conservativism | -0.01 | -0.03, 0.02 | .678 |
| Social conservativism | 0.14 | 0.11, 0.17 | <.001 |
| Subjective social status | -0.01 | -0.04, 0.01 | .380 |
| Importance of religion | 0.17 | 0.14, 0.20 | **<.001** |
| **Random Effects** |  | | |
| Residual | 0.79 | | |
| Country (intercept) | 0.10 | | |
| ICC | .11 | | |
| N (country) | 41 | | |
| Observations | 5766 | | |
| Marginal R^2^ / Conditional R^2^ | .116 / .213 | | |

*Note:* Gender was coded as male (1) and female (2).

# Supplementary Materials 3: Figures for Average Judgment Scores per Country


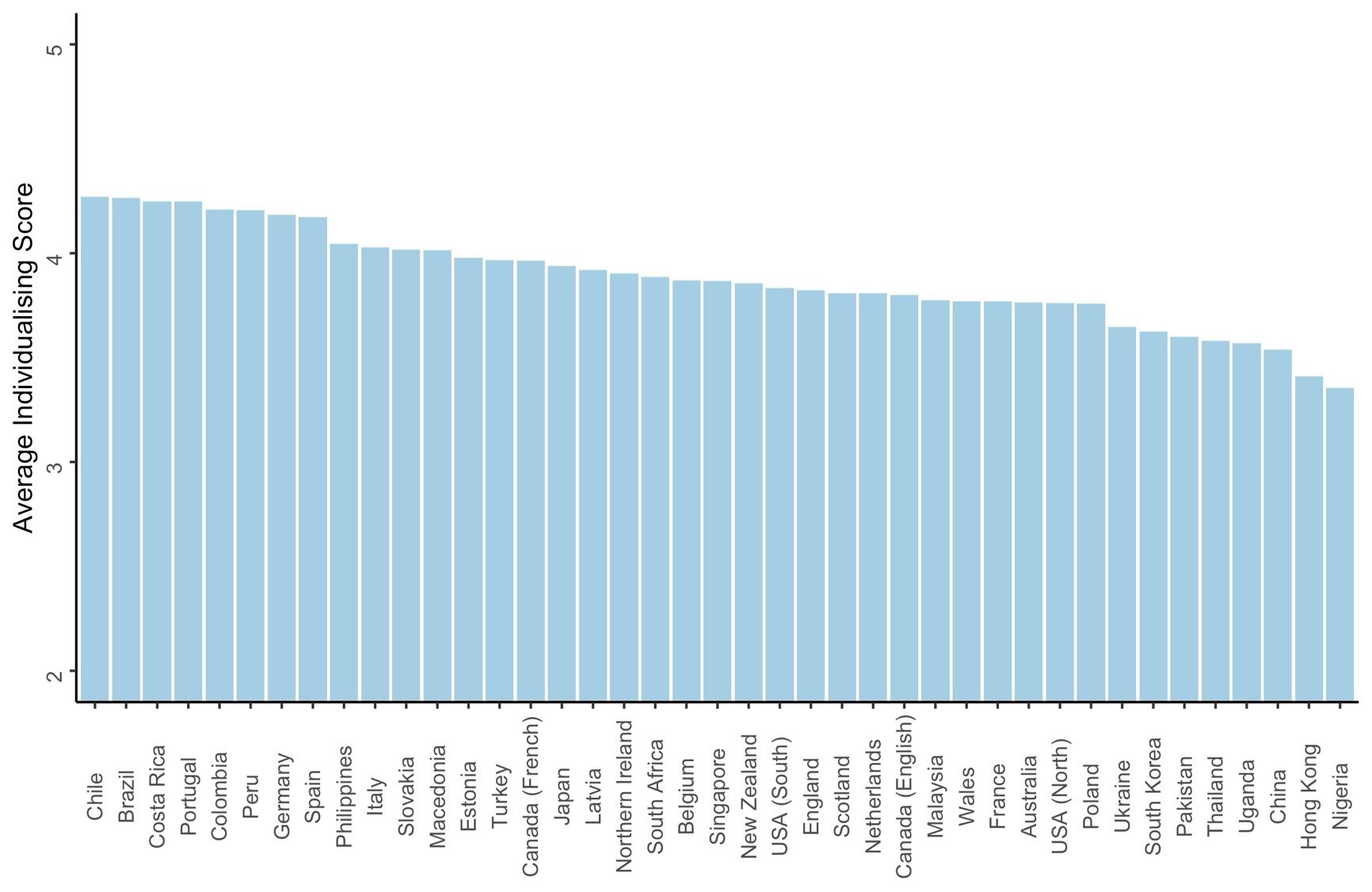


*Figure S1.* Average individualising judgment score per country.


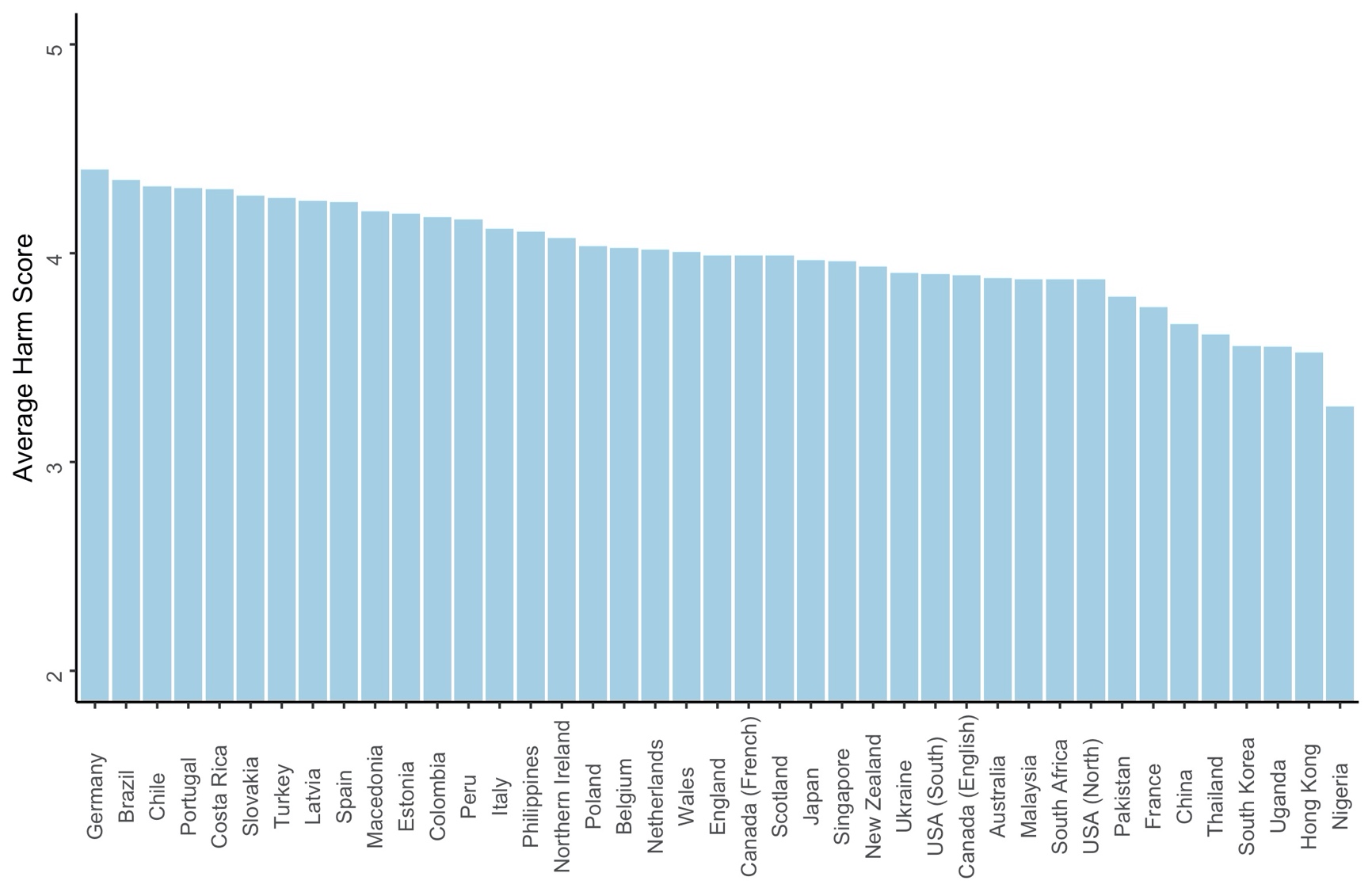


*Figure S2.* Average harm judgment score per country.


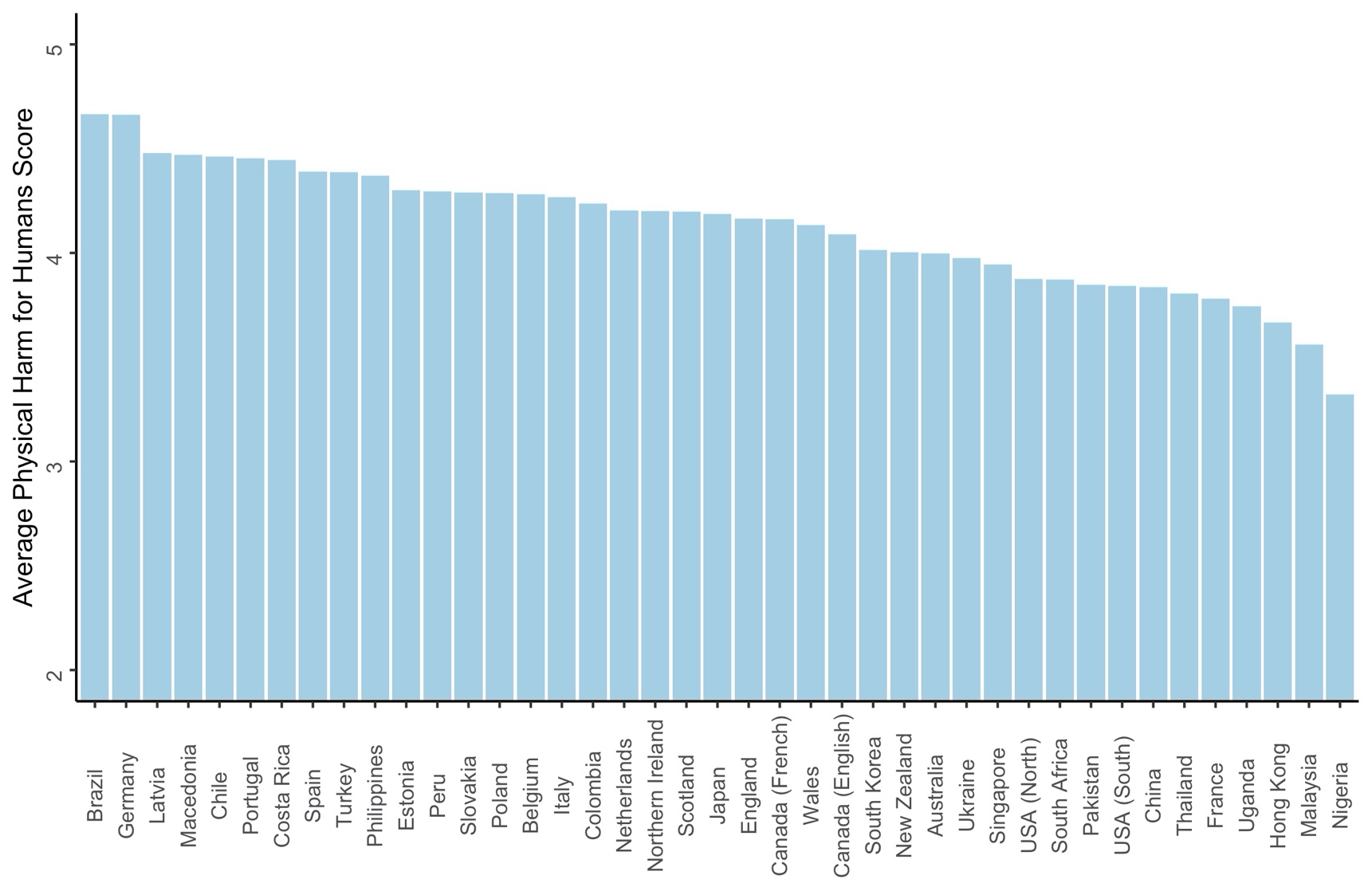


*Figure S3.* Average physical harm for humans judgment score per country.


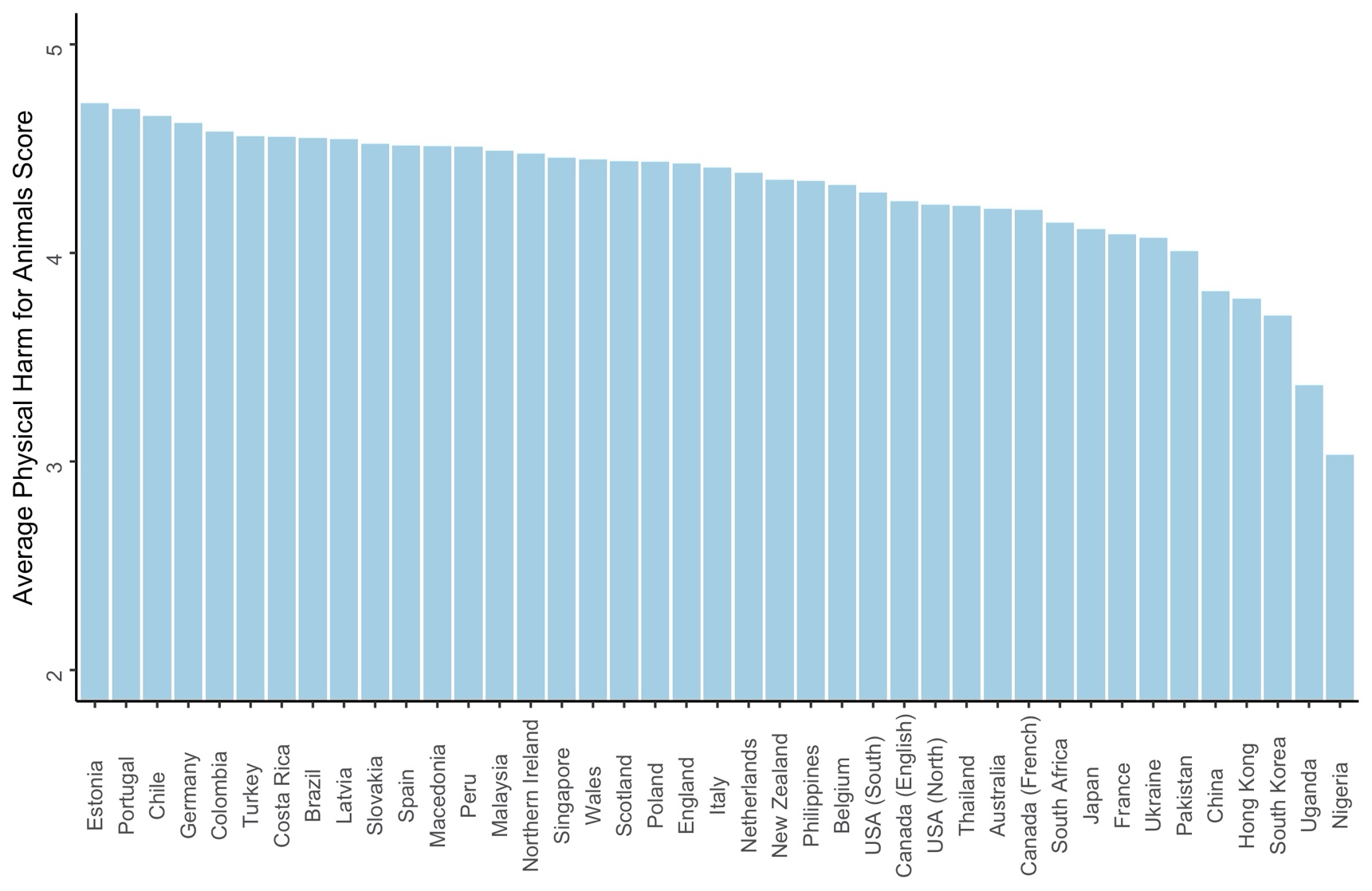


*Figure S4.* Average physical harm for animals judgment score per country.


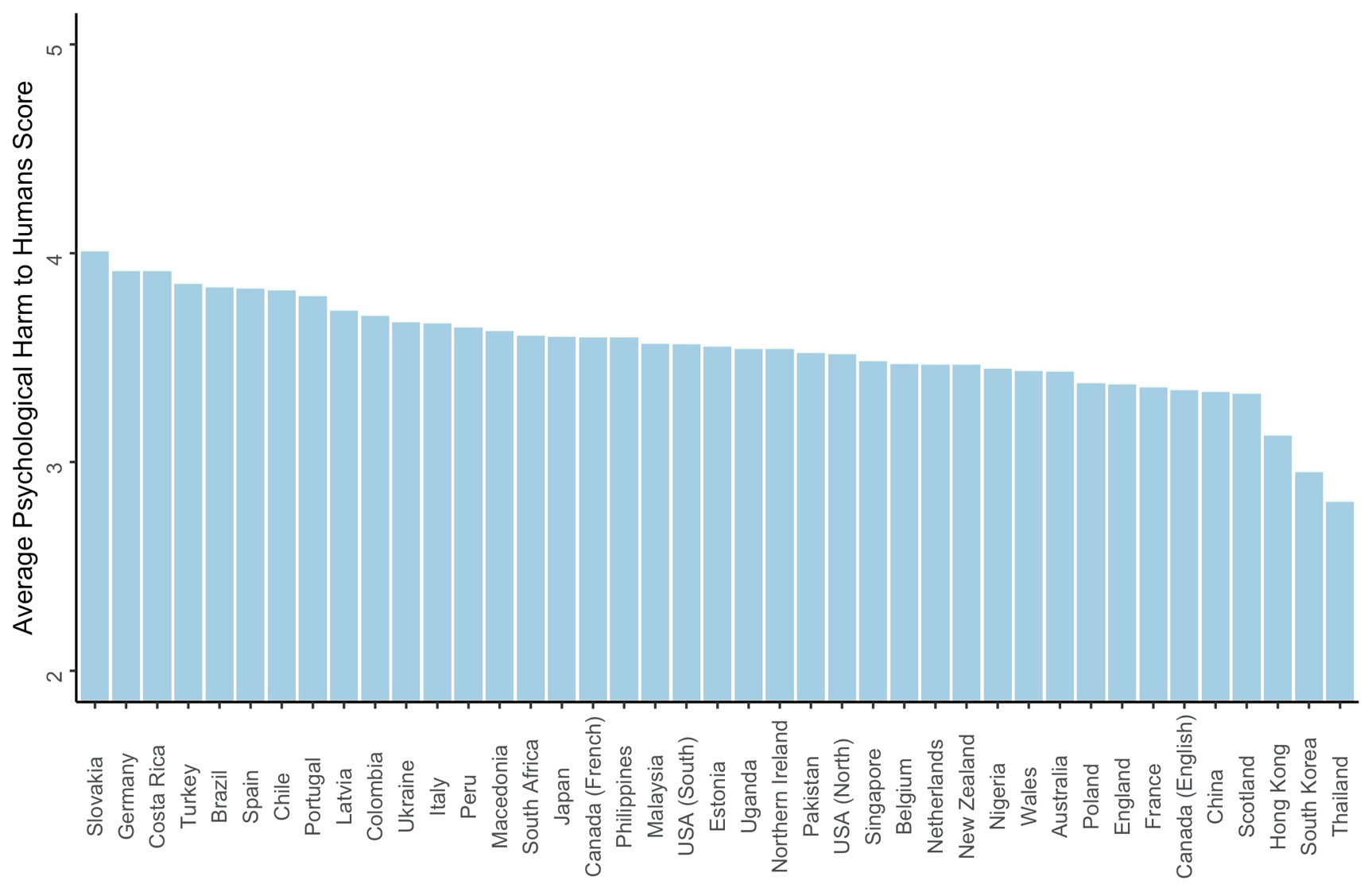


*Figure S5.* Average psychological harm for humans judgment score per country.


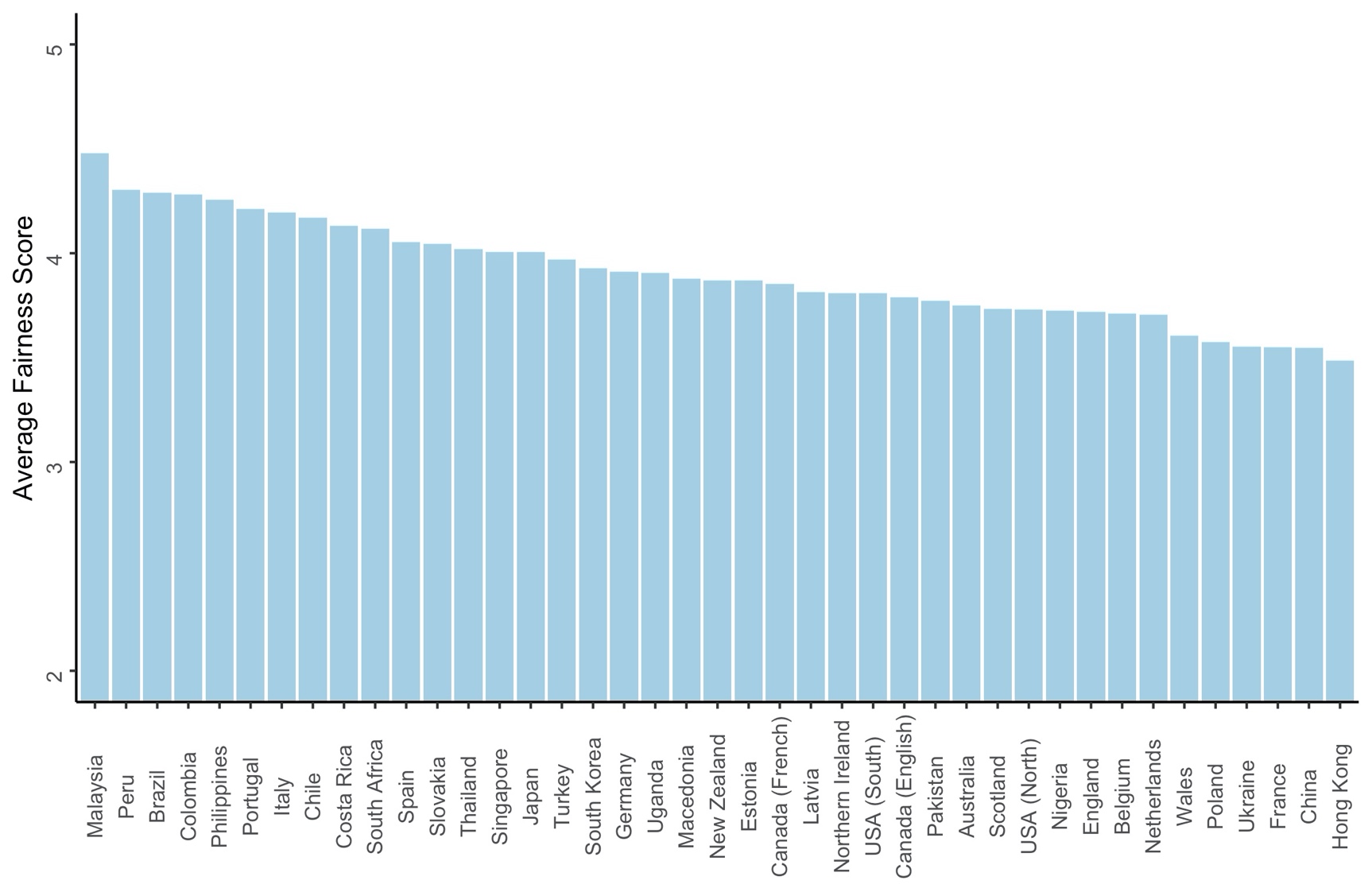


*Figure S6.* Average fairness judgment score per country.


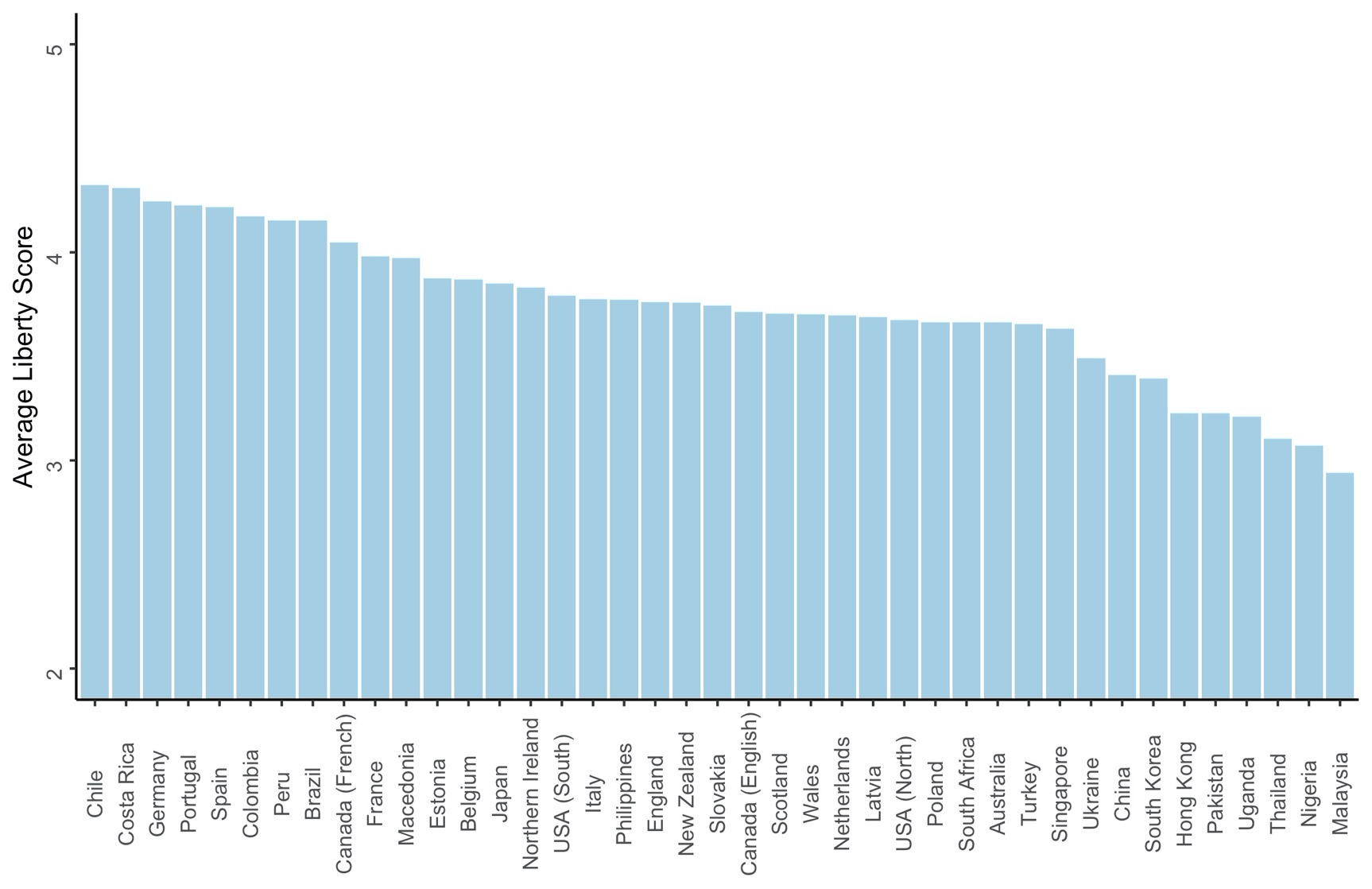


*Figure S7.* Average liberty judgment score per country.


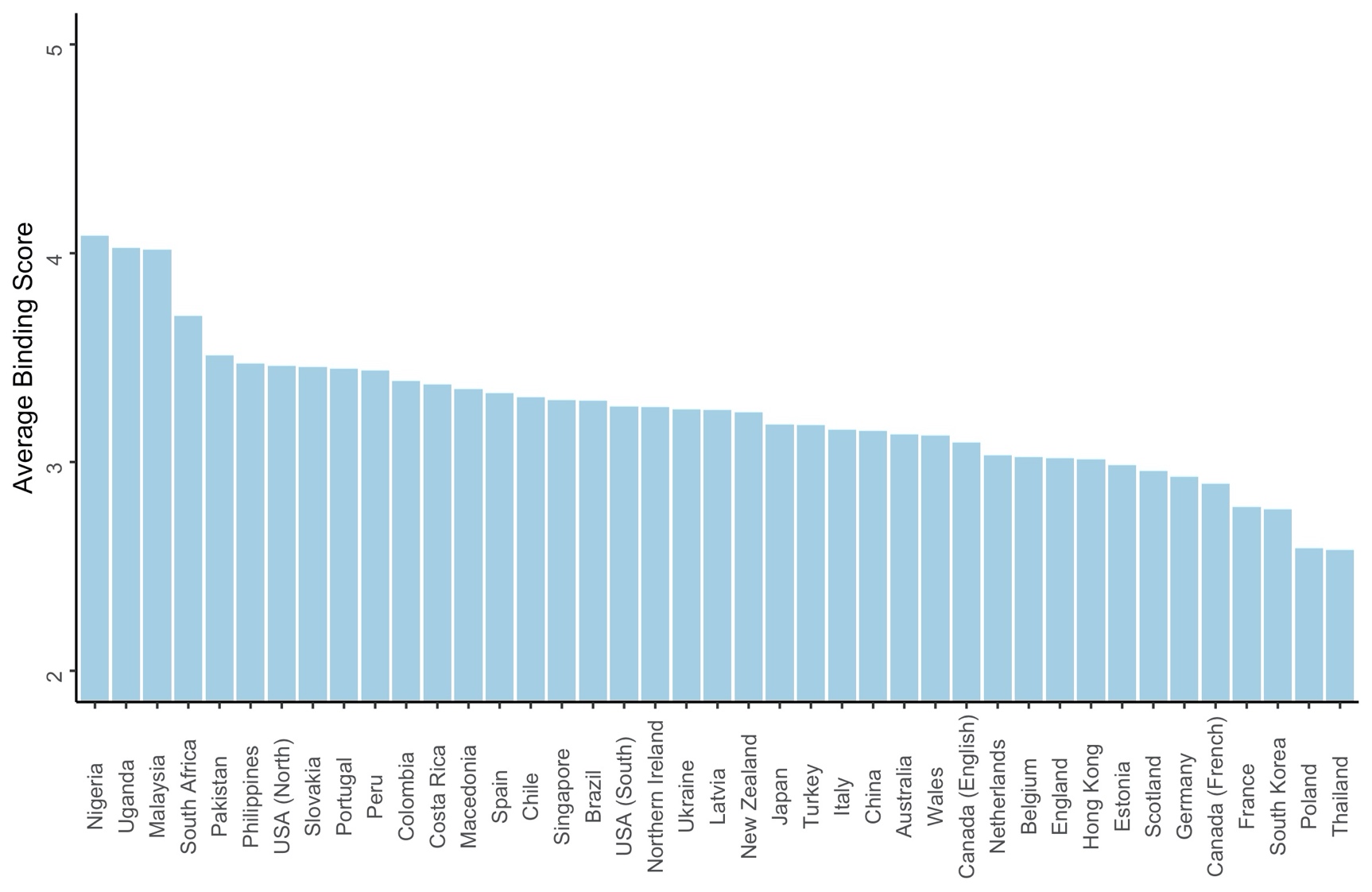


*Figure S8.* Average binding judgment score per country.


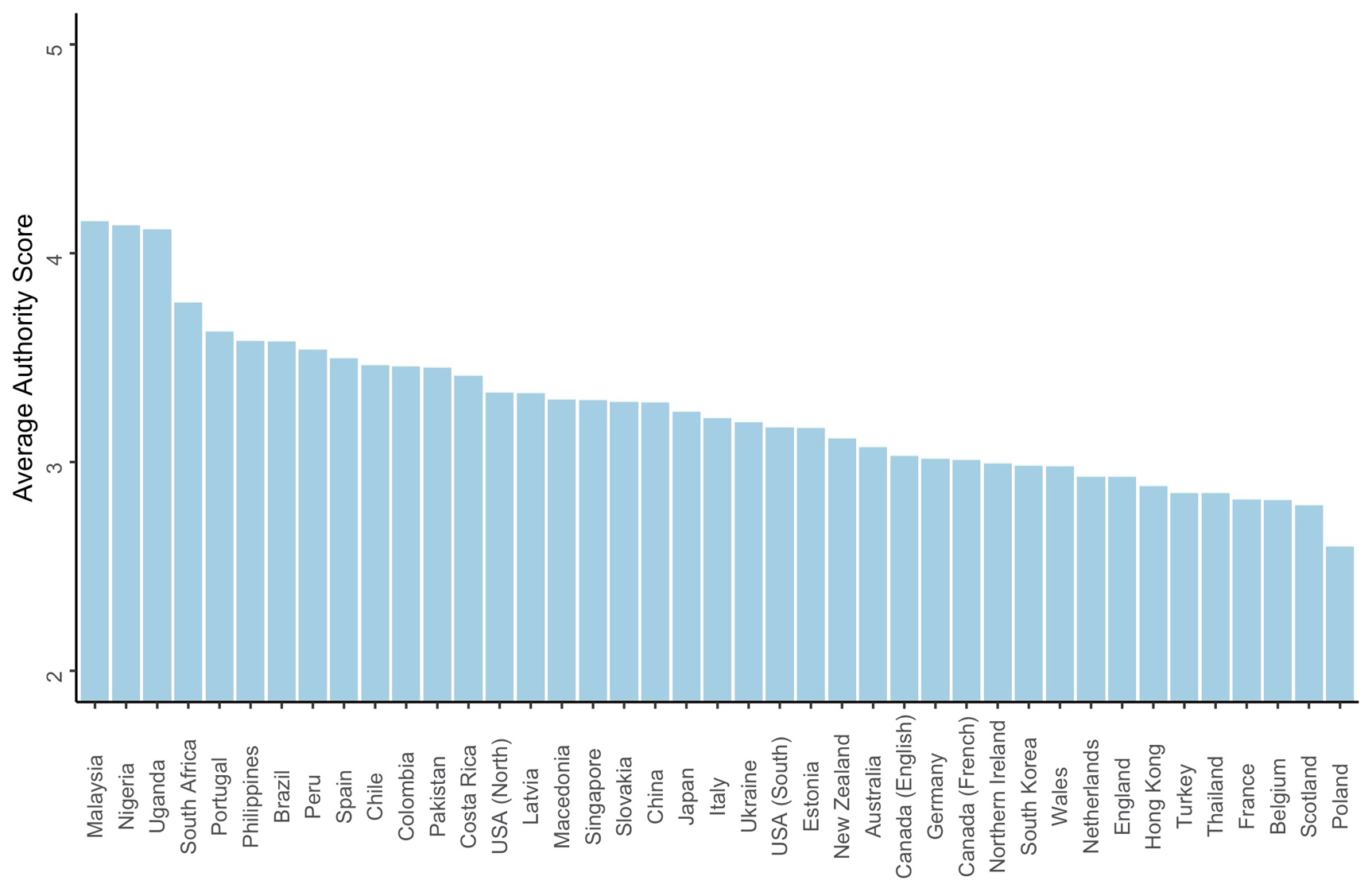


*Figure S9.* Average authority judgment score per country.


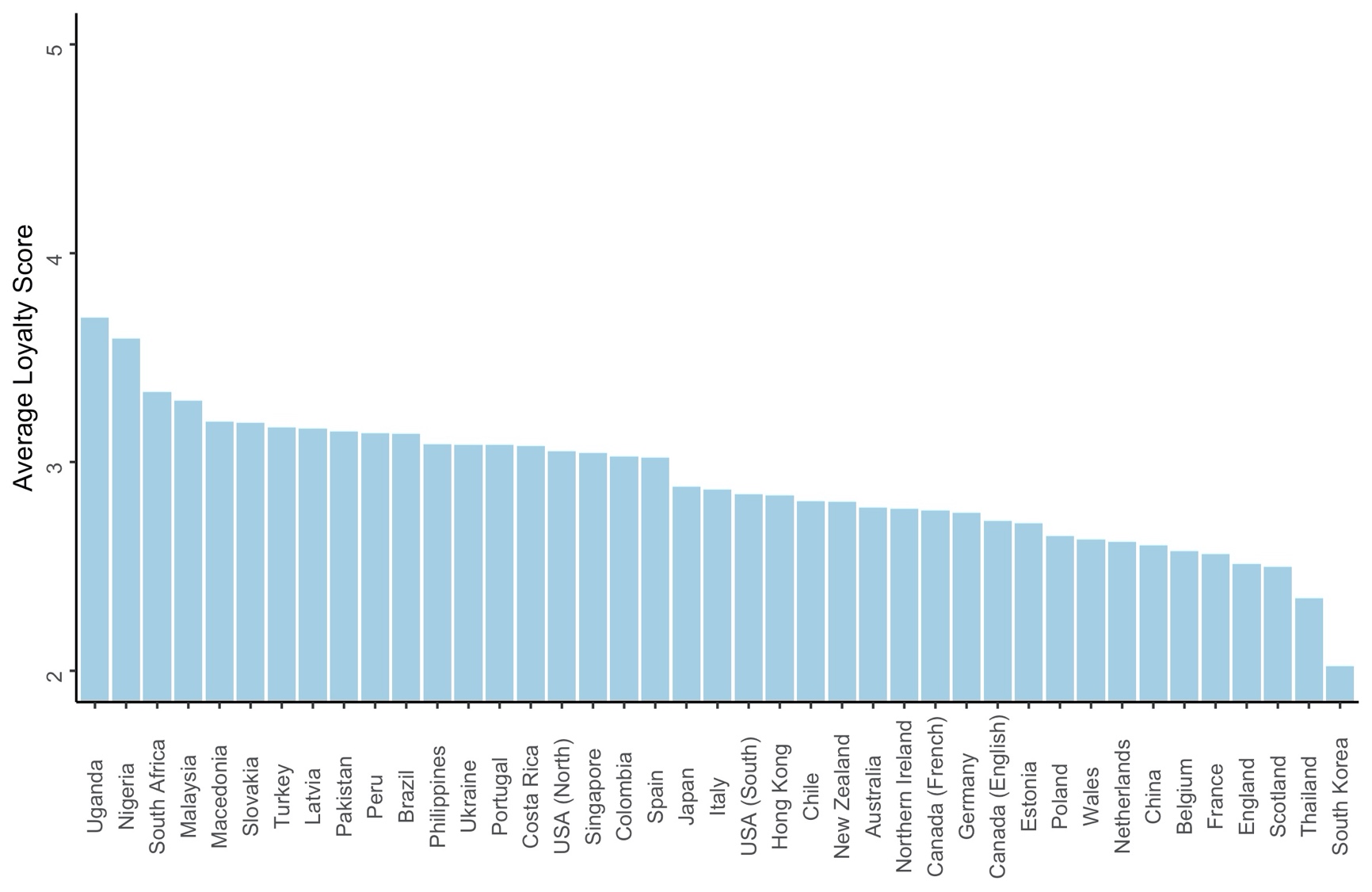


*Figure S10.* Average loyalty judgment score per country.


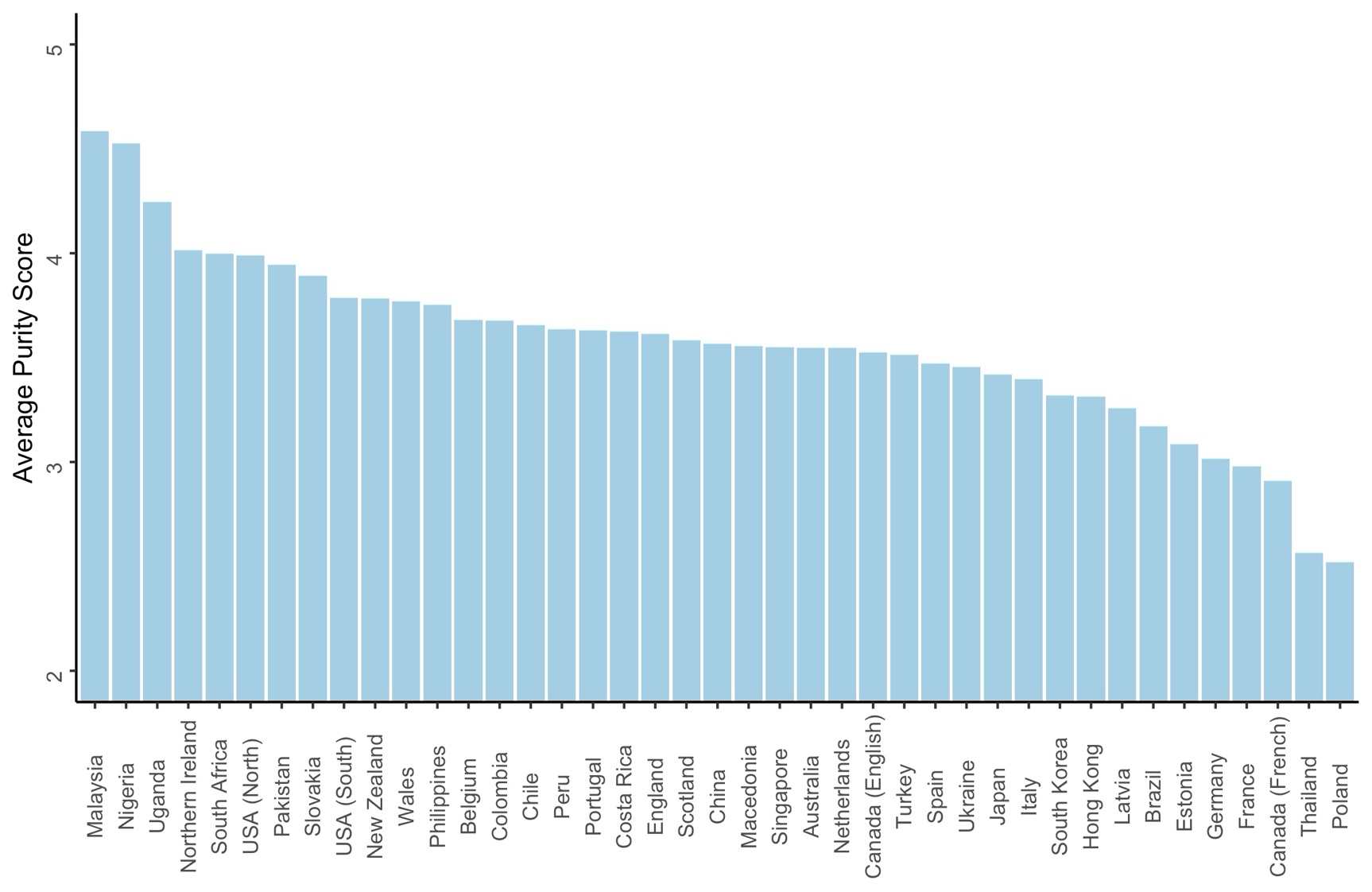


*Figure S11.* Average purity judgment score per country.

# Supplementary Materials 4: Correlations for Variables in Study 2

Table S51

*Correlations for Variables in Study 2*

|  | 1. Perceived Gini | 2. Gini (World Bank) | 3. Anomie in social fabric | 4. Harm | 5. Fairness | 6. Liberty | 7. Loyalty | 8. Authority | 9.Purity |
| --- | --- | --- | --- | --- | --- | --- | --- | --- | --- |
| 1. | – |  |  |  |  |  |  |  |  |
| 2. | 0.35*** | – |  |  |  |  |  |  |  |
| 3. | 0.20*** | 0.22*** | – |  |  |  |  |  |  |
| 4. | -0.03 | 0.02 | 0.11*** | – |  |  |  |  |  |
| 5. | 0.08*** | 0.18*** | 0.15*** | 0.46*** | – |  |  |  |  |
| 6. | -0.04* | 0.03* | 0.07*** | 0.50*** | 0.41*** | – |  |  |  |
| 7. | 0.19*** | 0.16*** | 0.12*** | 0.19*** | 0.31*** | 0.17*** | – |  |  |
| 8. | 0.20*** | 0.24*** | 0.13*** | 0.23*** | 0.45*** | 0.18*** | 0.52*** | – |  |
| 9. | 0.14*** | 0.12*** | 0.07*** | 0.14*** | 0.28*** | 0.05*** | 0.37*** | 0.45*** | – |

# Supplementary Materials 5: Mediation Results for Study 2

The mediation effect was generally replicated for all moral foundation judgments. However, it is worth noting these effects are small and on the cusp of significance.

Table S52

*Sixteen Linear Mixed Mediation Models Examining the Effect of Inequality Predictors on Moral Foundation Vignettes, via Anomie in the Social Fabric.*

|  |  | **Within-country effects** | | **Between-country effects** | |
| --- | --- | --- | --- | --- | --- |
| **Predictor** | **Outcome** | ***Indirect effect [95% CI]*** | ***Direct effect [95% CI]*** | ***Indirect effect [95% CI]*** | ***Direct effect [95% CI]*** |
| Perceived Gini | Individualizing | 0.003 [0.002, 0.01] | 0.03 [0.004, 0.05] | 0.01 [0.001, 0.02] | -0.08 [-0.24, 0.09] |
|  | Harm | 0.003 [0.002, 0.01] | 0.01 [-0.01, 0.04] | 0.01 [0.002, 0.02] | -0.12 [-0.28, 0.03] |
|  | Fairness | 0.003 [0.001, 0.01] | 0.01 [-0.01, 0.04] | 0.01 [0.001, 0.02] | 0.02 [-0.11, 0.15] |
|  | Liberty | 0.002 [0.001, 0.00] | 0.04 [0.01, 0.06] | 0.01 [0.001, 0.02] | -0.09 [-0.26, 0.08] |
|  | Binding | 0.002 [0.001, 0.00] | 0.03 [0.01, 0.06] | 0.01 [0.001, 0.01] | 0.25 [0.11, 0.38] |
|  | Purity | 0.002 [0.0002, 0.00] | 0.01 [-0.01, 0.04] | 0.004 [0.00, 0.01] | 0.21 [0.07, 0.35] |
|  | Authority | 0.002 [0.001, 0.00] | 0.02 [-0.005, 0.04] | 0.01 [0.00, 0.01] | 0.18 [0.05, 0.30] |
|  | Loyalty | 0.001 [-0.0001, 0.00] | 0.05 [0.02, 0.07] | 0.004 [-0.0003, 0.01] | 0.20 [0.09, 0.31] |
| Objective Gini | Individualizing | – | – | 0.01 [0.002, 0.02] | 0.16 [0.02, 0.30] |
|  | Harm | – | – | 0.01 [0.002,0.02] | 0.06 [-0.09, 0.20] |
|  | Fairness | – | – | 0.01 [0.002, 0.02] | 0.18 [0.06, 0.29] |
|  | Liberty | – | – | 0.01 [0.001, 0.02] | 0.15 [-0.001, 0.31] |
|  | Binding | – | – | 0.01 [0.001, 0.02] | 0.16 [0.03, 0.29] |
|  | Purity | – | – | 0.01 [0.001, 0.01] | 0.10 [-0.04, 0.23] |
|  | Authority | – | – | 0.01 [0.001, 0.01] | 0.18 [0.06, 0.29] |
|  | Loyalty | – | – | 0.004 [-0.0002, 0.01] | 0.10 [-0.004, 0.21] |

*Note.* Each line denotes a separate Linear Mixed Mediation Model, where both the within-country and between-country effects are included in the same model. Indirect effects were calculated for each of 1,000 bootstrapped samples, with the 95% confidence intervals calculated for the 2.5th and 97.5th percentiles. CI = confidence interval. Further details can be found in R code on OSF. Results will change slightly due to bootstrapping.

# Supplementary Materials 6: Amended Dictionary for Study 1

Words were sourced from the Moral Foundations Dictionary publicly available online: <https://moralfoundations.org/wp-content/uploads/files/downloads/moral%20foundations%20dictionary.dic>. Words were spot-checked on Twitter by entering them into the search function on Twitter which was ordered by most recent Tweets. If words did not bring up moral content (e.g., had various meanings, were not appropriately bringing up content from the specific foundation or were not commonly used on Twitter), they were removed from the final version of the dictionary. Below we first outline the words included, and the words that were removed from the final version of the dictionary

**Harm_Virtue**

safe*; peace*; compassion*; empath*; sympath*; care; caring; protect*; shield; shelter; secur*; benefit*; defen*; preserve

*Items removed:* amity; guard

**Harm_Vice**

harm*; suffer*; war; wars; warl*; warring; fight*; violen*; hurt*; kill; kills; killer*; killed; killing; endanger*; cruel*; brutal*; abuse*; damag*; ruin*; ravage; detriment*; crush*; attack*; annihilate*; destroy; abandon*; exploit; exploits; exploited; exploiting

*Items removed:* stomp; spurn; impair; wound*

**Fairness_Virtue**

fair; fairly; fairness; fairplay; fairminded; equal*; justice ; justness; justifi*; reciproc*; impartial*; egalitar*; rights; equity; unbias*; tolerant; unprejudice*; reasonable; honest*

*Items removed:* constant; fairmind* - changed to fairminded*; evenness; equivalent; equable; balance; homologous

**Fairness_Vice**

unfair*; unequal*; bias; biased; biasing; biases; unjust*; injust*; bigot; bigots; bigoted; discriminat*; disproportion*; inequitable; prejud*; dishonest; unscrupulous; favoritism; segregat*; exclusion; exclud*

*Items removed:* dissociate; preference

**Ingroup_Virtue**

segregat*; homeland*; family; families; familial; loyal*; patriot*; communal; commune*; communit*; communis*; comrad*; collectiv*; unite*; fellow*; solidarity; devot*; cliqu*; ally

*Items removed*: together; nation; group; cadre; joint; unison; guild; member; cohort; insider

**Ingroup_Vice**

abandon*; enem*; betray*; treason*; traitor*; treacher*; disloyal*; apostasy; apostate; deserted; deserter*; deserting; deceiv*; miscreant; terroris*; immigra*

*Items removed:* foreign*; individual*; jilt*; imposter; spy; sequester; renegade

**Authority_Virtue**

loyal*; obey*; obedien*; lawful*; duti*; honor*; respect; respectful*; respected; respects; hierarch*; authorit*; permission; bourgeoisie; caste*; complian*; command; allegian*; abide; revere*; venerat*; comply

*Items removed:* preserve; duty; law; legal*; order*; father*; mother; motherl*; mothering; mothers; tradition*; permit; status*; rank*; leader*; class; position; supremacy; control; submi*; serve; defere*; defer

**Authority_Vice**

betray*; treason*; traitor*; treacher*; disloyal*; apostasy; apostate; deserter*; deserting; defiant; dissent*; subver*; disrespect*; disobe*; sediti*; insubordinat*; illegal*; lawless*; insurgent; mutinous; defy*; dissident; defector; heretic*; nonconformist; protest; riot*; obstruct

*Items removed:* deserted; rebel*; agitat*; unfaithful; alienate; oppose; refuse; denounce; remonstrate

**Purity_Virtue**

preserve; piety; pious; purity; pure*; sacred*; chast*; holy; holiness; saint*; wholesome*; celiba*; virgin; virgins; virginity; virginal; integrity; modesty; abstinen*; unadulterated; maiden; virtuous; immaculate; innocent; pristine; church; churches

*Items removed:* clean*; steril*; holy; abstention; austerity; abstemiousness ; upright; limpid; decen*; refined

**Purity_Vice**

apostasy; apostate; disgust*; deprav*; disease*; unclean*; contagio*; indecen*; sinful*; sinner*; sins; sinned; sinning; slut*; whore; impiety; impious; profan*; gross; repuls*; promiscu*; adulter*; debauche*; prostitut*; unchaste; filth*; trashy; taint*; tarnish*; debase*; desecrat*; wicked*; pervert

*Items removed:* heretic*; exploit; exploits; exploited ; exploiting; ruin*; sin ; dirt*; sick*; lewd*; defile*; tramp; intemperate; wanton; profligate; obscen*; lax; stain*; blemish; wretched*; exploitat*

**Morality_General**

wretched*; wicked*; indecen*; integrity; lawful*; honest*; righteous*; moral*; ethic*; upstanding; goodness; principle*; blameless; praiseworthy; commendable; wrong*; evil; immoral*; offend*; offensive*; transgress*

*Items removed:* decen**;* upright; lesson; canon; doctrine; bad; good; character; proper; laudable; correct; legal*; value*; wholesome*; exemplary; worth*; ideal*; noble

# Supplementary Materials 7: Information About Multinational Sample in Study 2

**Table S53**

Countries, survey language, data collection method and total responses.

| **Country** | **Survey Format** | **Sample Size** | **Language** |
| --- | --- | --- | --- |
| Australia | Online | 386 | English |
| Belgium | Online | 225 | Dutch |
| Brazil | Online | 138 | Portuguese |
| Canada (English) | Online | 183 | English |
| Canada (French) | Online | 135 | French |
| Chile | Online | 69 | Spanish |
| China | Online | 235 | Chinese |
| Colombia | Online | 151 | Spanish |
| Costa Rica | Online | 130 | Spanish |
| England | Online | 149 | English |
| Estonia | Online | 63 | Estonian |
| France | Online | 191 | French |
| Germany | Online | 143 | German |
| Hong Kong | Online | 211 | English |
| Italy | Online | 222 | Italian |
| Japan | Online | 182 | Japanese |
| Latvia | Paper & Pencil | 120 | Latvian |
| Macedonia | Online | 124 | Macedonian |
| Malaysia | Online | 175 | Malay |
| Netherlands | Online | 110 | Dutch |
| New Zealand | Online | 134 | English |
| Nigeria | Paper & Pencil | 233 | English |
| Northern Ireland | Online | 110 | English |
| Pakistan | Online | 147 | English |
| Peru | Online | 200 | Spanish |
| Philippines | Online/Paper & Pencil | 151 | English |
| Poland | Paper & Pencil | 116 | Polish |
| Portugal | Online/Paper & Pencil | 137 | Portuguese |
| Scotland | Online | 110 | English |
| Singapore | Online | 102 | English |
| Slovakia | Paper & Pencil | 115 | Slovak |
| South Africa | Other(online) | 423 | English |
| South Korea | Other(online) | 127 | Korean |
| Spain | Online | 156 | Spanish |
| Thailand | Paper & Pencil | 99 | Thai |
| Turkey | Online | 275 | Turkish |
| Uganda | Online | 101 | English |
| Ukraine | Paper & Pencil | 110 | Ukrainian |
| USA (North) | Online | 181 | English |
| USA (South) | Online | 211 | English |
| Wales | Online | 85 | English |

# Supplementary Materials 8: Measures for Study 2

**Individual-Level Measures**

Moral Judgments

- Using the following scale, please indicate how morally wrong you consider the behaviours below.
  - Psychological harm
    - You see a girl laughing at another student forgetting her lines in a school play
    - You see a boy making fun of his brother for getting dumped by his girlfriend
    - You see a teenage girl openly staring at a disfigured woman as she walks past
  - Physical harm (animals)
    - You see a boy throwing rocks at cows that are grazing in the local pasture
    - You see a man lashing his pony with a whip for breaking loose from its pen
    - You see a boy setting a series of traps to kill stray cats in his neighbourhood
  - Physical harm (humans)
    - You see a boy placing a thumbtack sticking up on the chair of another student
    - You see a teacher hitting a student’s hand with a ruler for falling asleep in class
    - You see a woman spanking her child with a spatula for getting bad grades at school
  - Fairness
    - You see a tenant bribing a landlord to be the first to get their apartment repainted
    - You see an employee lying about how many hours she worked during the week
    - You see a politician using federal tax dollars to build an extension on his home
  - Liberty
    - You see a man forbidding his wife to wear clothing that he has not first approved
    - You see a boss pressuring employees to buy goods from her family’s general store
    - You see a father requiring his son to take up the family restaurant business
  - Authority
    - You see a teenage girl coming home late and ignoring her parents’ strict curfew
    - You see an employee trying to undermine all of her boss’ ideas in front of others
    - You see a student stating that her professor is a fool during an afternoon class
  - Loyalty
    - You see an employee joking with competitors about how bad his company did last year
    - You see a coach celebrating with the opposing team’s players who just won the game
    - You see a teacher publicly saying she hopes another school wins the math contest
  - Purity
    - You see a homosexual in a gay bar offering sex to anyone who buys him a drink
    - You see a man searching through the trash to find women’s discarded underwear

*Responses were recorded on a scale from 1 (not at all wrong) to 5 (extremely wrong).*

Country-level Gini

The Gini coefficient is one of the most frequently used indicators of income inequality. The scores range from 0 (perfect equality) to 1 (perfect inequality). As demonstrated in Figure S12a, the Lorenz curve reflects the distribution of wealth in a particular population, as determined by the amount of income the population possesses from poorest to richest (Osberg, 2017; Ramzai, 2020). The Gini coefficient is then calculated by dividing area A over (area A + area B). Area A reflects the area between the Lorenz curve and the line of equality whereas (area A + area B) reflects the entire area underneath the line of equality. Sourced from: <https://data.worldbank.org/indicator/SI.POV.GINI> (The World Bank, 2019b)

Perceived Gini Coefficient

The subjective Gini coefficient was calculated in an identical way to prior research (Kirkland et al., 2022; Sprong et al., 2019). Participants were asked to think of 100 citizens in their country. These 100 citizens were then to be placed in five categories: very poor, poor, average in wealth, wealthy and very wealthy. We then calculated the perceived Gini coefficient with the following four steps (see Figure S12b). These categories were first assigned the following units of wealth: 1, 2, 3, 4, and 5, respectively. Second, a cumulative wealth distribution histogram was calculated using the numbers each individual assigned to each category. The Lorenz curve and the line of equality were then drawn as demonstrated below. There were only five observations per participant (due to the five wealth categories), and the Lorenz curve was thus created by joining the diagonal of each column. The perceived Gini coefficient was then calculated by dividing area A over (area A + area B). The excel spreadsheet that automatically calculates the perceived Gini is available on OSF.


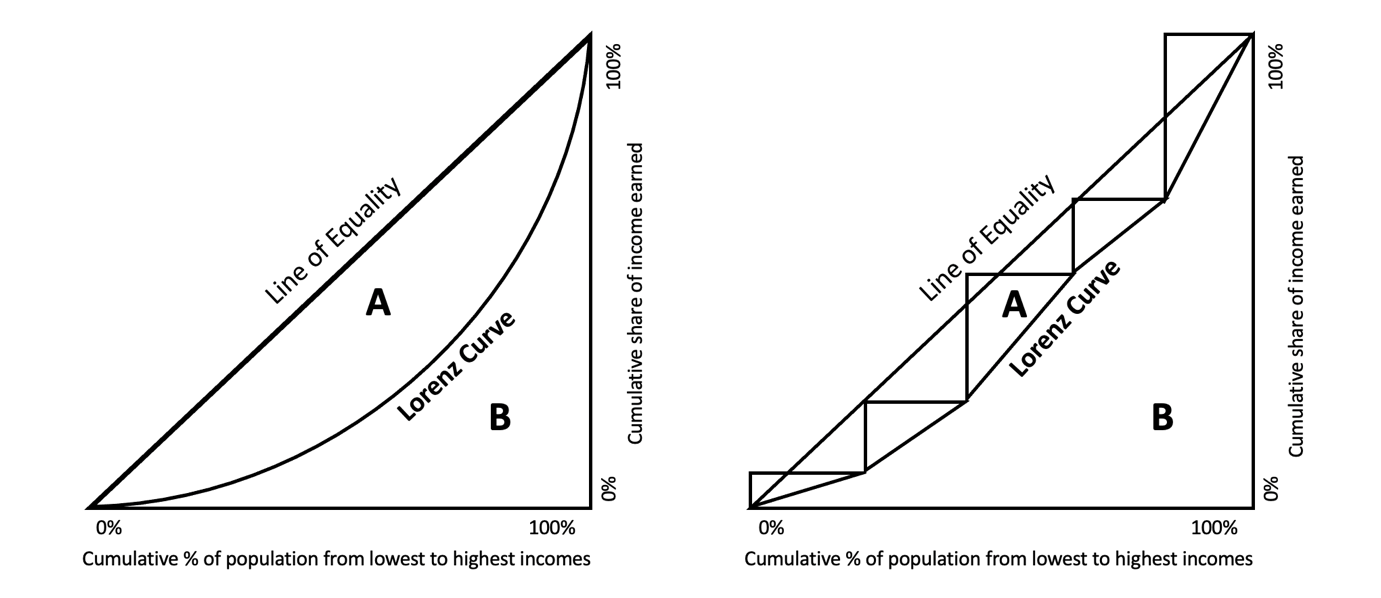


*Figure S12.* Calculation for the Gini coefficient (a) and perceived Gini coefficient (b). Figure adapted from Sprong, S., Jetten, J., Wang, Z., Peters, K., Mols, F., Verkuyten, M., Bastian, B., Ariyanto, A., Autin, F., Ayub, N., Badea, C., Besta, T., Butera, F., Costa-Lopes, R., Cui, L., Fantini, C., Finchilescu, G., Gaertner, L., Gollwitzer, M., … Wohl, M. J. A. (2019). “Our Country Needs a Strong Leader Right Now”: Economic Inequality Enhances the Wish for a Strong Leader. *Psychological Science*, *30*(11, Suppl.), 1625–1637. <https://doi.org/10.1177/0956797619875472>. Reprinted with permission.

Perceptions of anomie in the social fabric

- People think that there are no clear moral standards to follow
- Everyone thinks of himself/herself and does not help others in need
- Most of people think that if something works, it doesn’t really matter whether it is right or wrong
- People do not know who they can trust and rely on
- Most of the people think that honesty doesn’t work all the time; dishonesty is sometimes a better approach to get ahead
- People are cooperative – reverse coded

Age

- Age (in years): ______________

Gender

- Gender: ______________

Economic conservatism

- Please indicate your political beliefs from left/liberal to right/conservative on issues of the economy (e.g., social welfare, government spending, tax cuts):

*Responses were recorded on a scale from 1 (left/liberal) to 7 (right/conservative).*

Social conservatism

- Please indicate your political beliefs from left/liberal to right/conservative on social issues (e.g., immigration, homosexual marriage, abortion):

*Responses were recorded on a scale from 1 (left/liberal) to 7 (right/conservative)*

MacArthur Scale of Subjective Socioeconomic Status

- Think of this ladder as representing where people stand in <country>. At the top of the ladder are the people who have the most money, most education, and most respected jobs. At the bottom are the people who have the least money, least education, and least respected jobs or no job. The higher up you are on this ladder, the closer you are to the people at the very top, and the lower you are, the closer you are to the people at the very bottom. Where would you place yourself on this ladder? Please select a number corresponding to the rung where you think you stand at this time in your life, relative to other people in <country>.


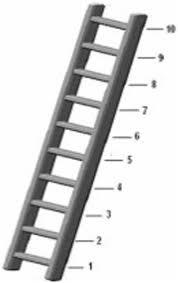


Responses were recorded on a scale from 1 (*least money, job prestige and education*) to 10 (*most money, job prestige and education*)

Importance of Religiosity

- If you do follow a religion, how important is that religion in your daily life?

*Responses were recorded on a scale from 1 (not at all important) to 7 (extremely important)*

GDP PPP

A measure of Gross Domestic Product per capita in international dollars. This measure adjusts for population size and accounts for the cost of living and inflation rates. Sourced from: <https://data.worldbank.org/indicator/NY.GDP.PCAP.PP.CD?end=2019&name_desc=false&start=1990&view=chart> (The World Bank, 2019a)

# Supplementary Materials 9: Wealth Gap Results for Study 2

There was a third variable in a dataset that measured participants’ perception of how large the wealth gap is between the rich and the poor:

- Now, we would like you to think of the poorest and the wealthiest people in <Country>. Overall, how large is the wealth gap between the poorest and the wealthiest people in <Country>?

*Responses were recorded on a scale from 1 (very small) to 7 (very large)*

Below we have outlined how this variable relates to moral judgments.

Table S54

*Linear Mixed Model Examining the Effect of Perceived Wealth Gap on Total Moral Judgment Score*

|  | **Total Moral Judgments** | | |
| --- | --- | --- | --- |
| *Predictors* | *Estimates* | *CI* | *p* |
| (Intercept) | -0.25 | -0.37, -0.13 | **<.001***** |
| Wealth gap (between-countries) | 0.05 | -0.08, 0.18 | .408 |
| Wealth gap (within-countries) | 0.12 | 0.09, 0.14 | **<.001***** |
| GDP | -0.08 | -0.21, 0.05 | .208 |
| Age | 0.02 | -0.00, 0.05 | .090 |
| Gender [female] | 0.36 | 0.31, 0.42 | **<.001***** |
| Economic conservativism | -0.04 | -0.06, -0.01 | **.011*** |
| Social conservativism | 0.06 | 0.03, 0.09 | **<.001***** |
| Subjective social status | -0.02 | -0.05, -0.00 | **.049*** |
| Importance of religion | 0.10 | 0.07, 0.13 | **<.001***** |
| **Random Effects** | | | |
| Residual | 0.81 | | |
| Country (intercept) | 0.13 | | |
| ICC | .14 | | |
| N (country) | 41 | | |
| Observations | 6016 | | |
| Marginal R^2^ / Conditional R^2^ | .065 / .193 | | |

*Note:* Gender was coded as male (1) and female (2).

Table S55

*Linear Mixed Model Examining the Effect of Perceived Wealth Gap on Individualising Moral Judgment Score*

|  | **Individualising** | | |
| --- | --- | --- | --- |
| *Predictors* | *Estimates* | *CI* | *p* |
| (Intercept) | -0.19 | -0.32, -0.07 | **.004**** |
| Wealth gap (between-countries) | 0.14 | 0.01, 0.28 | **.039*** |
| Wealth gap (within-countries) | 0.13 | 0.11, 0.16 | **<.001***** |
| GDP | 0.00 | -0.13, 0.14 | .975 |
| Age | 0.01 | -0.01, 0.04 | .385 |
| Gender [female] | 0.33 | 0.27, 0.38 | **<.001***** |
| Economic conservativism | -0.05 | -0.08, -0.02 | **<.001***** |
| Social conservativism | -0.02 | -0.05, 0.01 | .205 |
| Subjective social status | -0.00 | -0.03, 0.02 | .678 |
| Importance of religion | 0.01 | -0.02, 0.04 | .388 |
| **Random Effects** | | | |
| Residual | 0.76 | | |
| Country (intercept) | 0.14 | | |
| ICC | .16 | | |
| N (country) | 41 | | |
| Observations | 6035 | | |
| Marginal R^2^ / Conditional R^2^ | .071 / .215 | | |

*Note:* Gender was coded as male (1) and female (2).

Table S56

*Linear Mixed Model Examining the Effect of Perceived Wealth Gap on Binding Moral Judgment Score*

|  | **Binding** | | |
| --- | --- | --- | --- |
| *Predictors* | *Estimates* | *CI* | *p* |
| (Intercept) | -0.17 | -0.29, -0.06 | **.005**** |
| Wealth gap (between-countries) | -0.09 | -0.21, 0.03 | .148 |
| Wealth gap (within-countries) | 0.04 | 0.02, 0.06 | **<.001***** |
| GDP | -0.15 | -0.28, -0.03 | **.017*** |
| Age | 0.03 | 0.00, 0.06 | **.020*** |
| Gender [female] | 0.18 | 0.13, 0.23 | **<.001***** |
| Economic conservativism | 0.00 | -0.02, 0.03 | .826 |
| Social conservativism | 0.16 | 0.13, 0.19 | **<.001***** |
| Subjective social status | -0.04 | -0.06, -0.02 | **.001**** |
| Importance of religion | 0.18 | 0.15, 0.20 | **<.001***** |
| **Random Effects** | | | |
| Residual | 0.73 | | |
| Country (intercept) | 0.12 | | |
| ICC | .14 | | |
| N (country) | 41 | | |
| Observations | 6040 | | |
| Marginal R^2^ / Conditional R^2^ | .127 / .247 | | |

*Note:* Gender was coded as male (1) and female (2).

Table S57

*Linear Mixed Model Examining the Effect of Perceived Wealth Gap on Harm Moral Judgment Score*

|  | **Harm** | | |
| --- | --- | --- | --- |
| *Predictors* | *Estimates* | *CI* | *p* |
| (Intercept) | -0.24 | -0.37, -0.12 | **<.001***** |
| Wealth gap (between-countries) | 0.09 | -0.04, 0.23 | .159 |
| Wealth gap (within-countries) | 0.12 | 0.10, 0.15 | **<.001***** |
| GDP | -0.00 | -0.13, 0.13 | .984 |
| Age | -0.03 | -0.05, 0.00 | .066 |
| Gender [female] | 0.41 | 0.36, 0.46 | **<.001***** |
| Economic conservativism | -0.06 | -0.08, -0.03 | **<.001***** |
| Social conservativism | -0.03 | -0.05, 0.00 | .073 |
| Subjective social status | 0.00 | -0.02, 0.02 | .958 |
| Importance of religion | 0.02 | -0.01, 0.05 | .163 |
| **Random Effects** | | | |
| Residual | 0.76 | | |
| Country (intercept) | 0.14 | | |
| ICC | .15 | | |
| N (country) | 41 | | |
| Observations | 6049 | | |
| Marginal R^2^ / Conditional R^2^ | .073 / .214 | | |

*Note:* Gender was coded as male (1) and female (2).

Table S58

*Linear Mixed Model Examining the Effect of Perceived Wealth Gap on Fairness Moral Judgment Score*

|  | **Fairness** | | |
| --- | --- | --- | --- |
| *Predictors* | *Estimates* | *CI* | *p* |
| (Intercept) | -0.11 | -0.22, -0.00 | **.045*** |
| Wealth gap (between-countries) | 0.04 | -0.07, 0.16 | .474 |
| Wealth gap (within-countries) | 0.13 | 0.10, 0.15 | **<.001***** |
| GDP | -0.10 | -0.22, 0.01 | .076 |
| Age | 0.04 | 0.01, 0.06 | **.011*** |
| Gender [female] | 0.17 | 0.12, 0.23 | **<.001***** |
| Economic conservativism | -0.04 | -0.07, -0.01 | **.010*** |
| Social conservativism | 0.05 | 0.02, 0.08 | **.001**** |
| Subjective social status | -0.00 | -0.02, 0.02 | .986 |
| Importance of religion | 0.08 | 0.06, 0.11 | **<.001***** |
| **Random Effects** | | | |
| Residual | 0.82 | | |
| Country (intercept) | 0.10 | | |
| ICC | .11 | | |
| N (country) | 41 | | |
| Observations | 6057 | | |
| Marginal R^2^ / Conditional R^2^ | .052 / .153 | | |

*Note:* Gender was coded as male (1) and female (2).

Table S59

*Linear Mixed Model Examining the Effect of Perceived Wealth Gap on Liberty Moral Judgment Score*

|  | **Liberty** | | |
| --- | --- | --- | --- |
| *Predictors* | *Estimates* | *CI* | *p* |
| (Intercept) | -0.12 | -0.24, 0.01 | .066 |
| Wealth gap (between-countries) | 0.20 | 0.07, 0.33 | **.004**** |
| Wealth gap (within-countries) | 0.08 | 0.05, 0.10 | **<.001***** |
| GDP | 0.10 | -0.03, 0.23 | .130 |
| Age | 0.01 | -0.01, 0.04 | .348 |
| Gender [female] | 0.22 | 0.16, 0.27 | **<.001***** |
| Economic conservativism | -0.03 | -0.05, 0.00 | .056 |
| Social conservativism | -0.06 | -0.09, -0.04 | **<.001***** |
| Subjective social status | -0.01 | -0.03, 0.01 | .390 |
| Importance of religion | -0.07 | -0.09, -0.04 | **<.001***** |
| **Random Effects** | | | |
| Residual | 0.77 | | |
| Country (intercept) | 0.14 | | |
| ICC | .15 | | |
| N (country) | 41 | | |
| Observations | 6052 | | |
| Marginal R^2^ / Conditional R^2^ | .083 / .221 | | |

*Note:* Gender was coded as male (1) and female (2).

Table S60

*Linear Mixed Model Examining the Effect of Perceived Wealth Gap on Authority Moral Judgment Score*

|  | **Authority** | | |
| --- | --- | --- | --- |
| *Predictors* | *Estimates* | *CI* | *p* |
| (Intercept) | -0.16 | -0.27, -0.06 | **.004**** |
| Wealth gap (between-countries) | -0.05 | -0.16, 0.07 | .415 |
| Wealth gap (within-countries) | 0.04 | 0.02, 0.06 | **<.001***** |
| GDP | -0.20 | -0.32, -0.09 | **.001**** |
| Age | 0.04 | 0.01, 0.07 | **.003**** |
| Gender [female] | 0.19 | 0.14, 0.24 | **<.001***** |
| Economic conservativism | -0.00 | -0.03, 0.02 | .804 |
| Social conservativism | 0.13 | 0.10, 0.16 | **<.001***** |
| Subjective social status | -0.06 | -0.09, -0.04 | **<.001***** |
| Importance of religion | 0.15 | 0.13, 0.18 | **<.001***** |
| **Random Effects** | | | |
| Residual | 0.73 | | |
| Country (intercept) | 0.10 | | |
| ICC | .12 | | |
| N (country) | 41 | | |
| Observations | 6053 | | |
| Marginal R^2^ / Conditional R^2^ | .127 / .234 | | |

*Note:* Gender was coded as male (1) and female (2).

Table S61

*Linear Mixed Model Examining the Effect of Perceived Wealth Gap on Loyalty Moral Judgment Score*

|  | **Loyalty** | | |
| --- | --- | --- | --- |
| *Predictors* | *Estimates* | *CI* | *p* |
| (Intercept) | -0.08 | -0.18, 0.02 | .108 |
| Wealth gap (between-countries) | -0.03 | -0.13, 0.08 | .603 |
| Wealth gap (within-countries) | -0.01 | -0.04, 0.01 | .301 |
| GDP | -0.14 | -0.24, -0.04 | **.009**** |
| Age | 0.09 | 0.06, 0.11 | **<.001***** |
| Gender [female] | 0.07 | 0.02, 0.13 | **.008**** |
| Economic conservativism | 0.01 | -0.01, 0.04 | .339 |
| Social conservativism | 0.10 | 0.07, 0.13 | **<.001***** |
| Subjective social status | -0.02 | -0.05, 0.00 | .066 |
| Importance of religion | 0.09 | 0.06, 0.12 | **<.001***** |
| **Random Effects** | | | |
| Residual | 0.86 | | |
| Country (intercept) | 0.08 | | |
| ICC | .08 | | |
| N (country) | 41 | | |
| Observations | 6052 | | |
| Marginal R^2^ / Conditional R^2^ | .063 / .139 | | |

*Note:* Gender was coded as male (1) and female (2).

Table S62

*Linear Mixed Model Examining the Effect of Perceived Wealth Gap on Purity Moral Judgment Score*

|  | **Purity** | | |
| --- | --- | --- | --- |
| *Predictors* | *Estimates* | *CI* | *p* |
| (Intercept) | -0.16 | -0.28, -0.05 | **.006**** |
| Wealth gap (between-countries) | -0.13 | -0.25, -0.01 | **.038*** |
| Wealth gap (within-countries) | 0.07 | 0.04, 0.09 | **<.001***** |
| GDP | -0.04 | -0.16, 0.08 | .491 |
| Age | -0.04 | -0.07, -0.02 | **.002**** |
| Gender [female] | 0.16 | 0.11, 0.21 | **<.001***** |
| Economic conservativism | -0.01 | -0.03, 0.02 | .641 |
| Social conservativism | 0.15 | 0.12, 0.18 | **<.001***** |
| Subjective social status | -0.01 | -0.03, 0.01 | .385 |
| Importance of religion | 0.18 | 0.15, 0.20 | **<.001***** |
| **Random Effects** | | | |
| Residual | 0.79 | | |
| Country (intercept) | 0.11 | | |
| ICC | .12 | | |
| N (country) | 41 | | |
| Observations | 6056 | | |
| Marginal R^2^ / Conditional R^2^ | .100 / .208 | | |

*Note:* Gender was coded as male (1) and female (2).

# References

Gelfand, M. J., Raver, J. L., Nishii, L., Leslie, L. M., Lun, J., Lim, B. C., Duan, L., Almaliach, A., Ang, S., Arnadottir, J., Aycan, Z., Boehnke, K., Boski, P., Cabecinhas, R., Chan, D., Chhokar, J., D’Amato, A., Ferrer, M., Fischlmayr, I. C., … Yamaguchi, S. (2011). Differences between tight and loose cultures: A 33-nation study. *Science*, *332*(6033), 1100–1104. https://doi.org/10.1126/science.1197754

Goodman, E., Adler, N. E., Kawachi, I., Frazier, A. L., Huang, B., & Colditz, G. A. (2001). Adolescents’ perceptions of social status: Development and evaluation of a new indicator. *Pediatrics*, *108*(2), 1–8. https://doi.org/10.1542/peds.108.2.e31

Kirkland, K., Crimston, C. R., Jetten, J., Rudnev, M., Acevedo-triana, C., Amiot, C. E., Ausmees, L., Baguma, P., Barry, O., Becker, M., Bilewicz, M., Boonyasiriwat, W., Castelain, T., Costantini, G., Dimdins, G., Espinosa, A., Finchilescu, G., Fischer, R., Friese, M., … Bastian, B. (2022). Moral expansiveness around the world: The role of societal factors across 36 countries. *Social Psychological and Personality Science*, *14*(3), 305–318. https://doi.org/10.1177/19485506221101767

Osberg, L. (2017). On the limitations of some current usages of the Gini Index. *Review of Income and Wealth*, *63*(3), 574–584. https://doi.org/10.1111/roiw.12256

Ramzai, J. (2020). *Clearly Explained: Gini coefficient and Lorenz curve*. Towards Data Science.

Sprong, S., Jetten, J., Wang, Z., Peters, K., Mols, F., Verkuyten, M., Bastian, B., Ariyanto, A., Autin, F., Ayub, N., Badea, C., Besta, T., Butera, F., Costa-Lopes, R., Cui, L., Fantini, C., Finchilescu, G., Gaertner, L., Gollwitzer, M., … Wohl, M. J. A. (2019). “Our country needs a strong leader right now”: Economic inequality enhances the wish for a strong leader. *Psychological Science*, *30*(11), 1625–1637. https://doi.org/10.1177/0956797619875472

The World Bank. (2019a). *GDP per capita, PPP (current international $)*. https://data.worldbank.org/indicator/NY.GDP.PCAP.PP.CD?end=2019&name_desc=false&start=1990&view=chart

The World Bank. (2019b). *Gini index (World Bank estimate)*. https://data.worldbank.org/indicator/SI.POV.GINI
